# Computed tomography anatomy of the pony thorax and abdomen and an example of a clinical application

**DOI:** 10.1186/s13028-026-00891-4

**Published:** 2026-11-03

**Authors:** Péter Tóth, Bence Földes, Emese Bódai, Zsófia Pál, Mátyás Kapiller

**Affiliations:** 1 Department and Clinic of Equine Medicine at the University of Veterinary Medicine Budapest, Dóra major, Üllő, 2225 Hungary; 2 Greenmount Equine Hospital, Patrickswell, V94 VPX7 Ireland; 3 Department of Anatomy and Histology at the University of Veterinary Medicine Budapest, Budapest, 1078 Hungary

**Keywords:** Abdomen, Body size, Computed tomography, Diagnostic tools, Horses, Large gantry, Thorax

## Abstract

**Background:**

In veterinary medicine, computed tomography (CT) is a valuable diagnostic tool that provides detailed cross-sectional images with higher contrast resolution than conventional radiography. However, owing to the large body-size of horses, CT has been primarily limited in equine veterinary practice to imaging of the head, neck, and extremities, as imaging studies of the thoracic and abdominal cavities are not possible with standard gantry size in medium-to-large horses. In this study, we aimed to characterise the normal thoracic and abdominal anatomy of ponies using CT and to describe an example of the potential clinical applications of abdominal CT. CT examination of two ponies was performed in dorsal recumbency using a helical 16-slice multidetector scanner with a 90 cm-diameter gantry. The normal anatomy of these cavities was described and compared with anatomical cross-sections, and a clinical case involving colic surgery was analysed.

**Results:**

Most of the thoracic and abdominal organs in the anatomical section were identifiable, and the use of these images as a reference assisted well in the interpretation of the clinical case scans. The advanced imaging technique aided in surgical planning. In the case involving colic surgery, no evidence of thick-walled intestinal segments was found on CT, but contents including gas, fluid, different types of ingests, and sand were identified preoperatively. Nerves or serous membranes (except for the spinal cord) could not be clearly identified on native CT images.

**Conclusions:**

CT was useful for clinical diagnosis, but the small number of cases included in the study is a limitation that should be acknowledged.

**Supplementary Information:**

The online version contains supplementary material available at https://doi.org/10.1186/s13028-026-00891-4.

## Background

Computed tomography (CT) has become a valuable diagnostic tool in veterinary medicine, providing detailed cross-sectional images with superior contrast resolution that surpasses conventional radiography [1]. Considering the large body-size of horses, the use of CT in equine veterinary practice has been primarily limited to imaging of the head, neck, and extremities [1, 2]. However, advances in CT technology, including the development of large-gantry systems, now enable thoracic and abdominal imaging in foals, ponies, as well as small-to-medium size horses [1, 3, 4].

The first veterinary application of CT, reported in the 1980s, involved the examination of the central nervous system of dogs [2]. Kraft and Gavin [5] found that the first CT scans of horses were performed in 1986, concurring with the introduction of magnetic resonance imaging (MRI) scans.

Whole-body CT anatomical atlases exist for several species, such as houndsharks [6] and dogs [7, 8] but has not been attempted in horses.

Previous studies have reported imaging data of the head [9], neck [10], stifle [11], hock [12], fetlock [13], foot [14], and groin area [15] of adult horses, as well as of foals [16–18]. Two studies on thoracic CT in ponies have been reported [19, 20], as well as a case report involving enterolithiasis [21]. Only two abdominal CT studies in adult horses of normal size have been reported [3, 4].

Traditional diagnostic modalities of the abdominal and thoracic cavities, such as ultrasonography and radiography, provide valuable information; however, they often fail to provide visual information on deeper structures, subtle lesions, or complex anatomical regions [22–24]. Conversely, CT offers superior soft-tissue contrast and three-dimensional reconstructions, making it a potentially valuable tool for evaluating pulmonary, cardiovascular, gastrointestinal, and other internal disorders in horses [25]. In humans and small animals, CT with normal size gantry [26] is routinely used for both abdominal and thoracic scans [1], however the use of large bore CT scanners in thoracic and abdominal imaging of horses remains relatively underexplored owing to challenges such as patient positioning and the requirement for general anaesthesia.

Abdominal radiography in horses is mainly limited by their large body size, preventing adequate X-ray penetration to visualise deep abdominal structures. The thick abdominal wall and overlapping organs result in poor contrast and image resolution. Additionally, gas and ingesta within the intestines can obscure details, making the diagnosis of specific conditions difficult without more advanced imaging techniques [23].

Abdominal ultrasonography in horses is also primarily limited by their large size and the depth of the abdominal organs, which can hinder access and result in poor image resolution. Moreover, gas-filled intestines obstruct sound waves, reducing the visibility of deeper structures. Additionally, the thick body wall and limited acoustic windows make it challenging to obtain comprehensive views of all abdominal organs [24].

Abdominal CT examination provides detailed cross-sectional images of the abdomen, allowing surgeons to visualise organ structures, masses, and vascular anatomy, and to precisely describe relationships between tissues. This advanced imaging technique can help identify the exact location, size, and extent of lesions or abnormalities, allowing for a more accurate diagnosis and surgical planning [1]. CT may provide additional diagnostic information to evaluate these regions; for instance, a 90-cm gantry can image small-to-medium size horses (≤460 kg) [3].

CT windowing adjusts image contrast to highlight specific tissues, with soft tissue and bone windows being the most used settings. Soft tissue windows enhance the visibility of organs, fat, and vessels, making them ideal for evaluating abdominal and thoracic organs, including the intestines, liver, heart, and lungs. Conversely, bone windows increase the contrast of dense structures such as bones, making fractures or calcifications more visible but obscuring soft tissues. It is thus essential to use the correct window because an inappropriate setting can make important findings either invisible or unclear. In abdominal and thoracic CT examinations, radiologists typically review images using multiple windows to capture critical details. In CT imaging, filters (reconstruction kernels) are mathematical processes that determine the sharpness and smoothness of the reconstructed image. Sharp filters improve edge detail but increase noise, while smooth filters reduce noise and are more suited for the evaluation of soft tissues. Appropriate windowing and filters are important in CT because they optimize image contrast and sharpness for accurate image interpretation [27].

CT measures and computes the spatial distribution of the linear attenuation coefficient in each pixel. However, this coefficient cannot be used for image interpretation and comparison. CT values characterize the linear attenuation coefficient of the tissue in each volume element relative to the attenuation value of water. CT values (CT numbers) are specified in Hounsfield Unit (HU) [28]. CT numbers have been reported for horses in studies focused in orthopaedics [29] and in dentistry [30], but not for abdominal parenchymal organs or bowel contents.

In this study, we aimed to provide insights into the role of CT in the diagnosis of thoracoabdominal pathologies. Moreover, we analysed a clinical case in order to determine which organs and intestinal contents can be visualised under physiological conditions, and to identify the limitations of the imaging modality. Therefore, we analysed CT images corresponding to anatomical sections in order to identify anatomical structures. Our primary focus was on the thoracic and abdominal organs; however, bony structures and trunk muscles were also identified. We anticipate that our study will contribute to the better understanding of equine thoracic and abdominal CT imaging, and serve as a reference for future research as well as an immediate aid for clinicians.

## Methods

We conducted an anatomical study correlating CT images with anatomical sections, and present an example of the clinical application of abdominal CT using two Shetland ponies. The first was a 12-year-old mare weighing 130 kg. It was euthanised due to animal welfare issues owing to an open metatarsal fracture after the owner declined surgical treatment. According to the owner, it had no shown any respiratory or colic symptoms during the previous 6 months. No pathological changes were found in either the thoracic or abdominal region during the clinical examination.

The CT examination was performed post mortem immediately after euthanasia at the Department and Clinic of Equine Medicine of the University of Veterinary Medicine Budapest at Üllő, Hungary; using a Canon Toshiba Aquilion Prime SP 16-slice helical multidetector scanner with a 90 cm gantry diameter. Scan parameters included a 2-mm slice thickness, a 135 kV and 450 mA exposure, and a 0.75 s rotation time. Images were acquired using both smooth and sharp algorithms, and the image files obtained using the smooth algorithm were used for the analysis. The cadaver was scanned in two segments with a 70-cm acquisition field of view (FOV) in dorsal recumbency.in soft tissue algorithm. Images were viewed with the WW (window width) of 250 and the WL (window length) of 50 and with matrix 512 × 512 (Additional file 1). After CT imaging, the specimen was transferred to the −18 °C freezing chamber from the Department and Clinic of Equine Medicine at Üllő, Hungary. During transportation and placement, the exact position in which the pony was fixed for the CT scans was maintained as accurately as possible. For this purpose, the body was placed in a standard semi-circular bin cut in a half lengthwise and secured with ratchet straps (Additional file 1).

The cadaver (frozen for three months) was transported to the Department of Anatomy and Histology at the University of Veterinary Medicine Budapest, Hungary, for transverse sectioning into 39 slices (each with a thickness of 2 cm) starting from the manubrium sterni to the rectum, using a Reich Plastination Band Saw HL-1430. Each frozen slice was placed with its caudal surfaces facing upward and wiped with ethyl alcohol (Bradoderm hand sanitiser containing 65% ethyl alcohol, Florin Chemical and Trading Ltd., Szeged, Hungary) to remove residual frozen tissue debris from sectioning. Based on our experience, we found that ethyl alcohol provided superior cleaning and allowed for sharper and clearer images. Photographs of the prepared slices were subsequently captured using a Nikon D850 camera body with an AF-S Nikkor 24–70 mm 1:2.8E ED VR lens. Fixatives were not used for tissue preservation; only freezing was applied to maintain maximal anatomical integrity.

The second case involved a 9-year-old pony mare weighing 125 kg, presenting with suspected large colon impaction containing a large amount of fluid and sand as well. The pony had been kept on sandy area where it fed from the ground, showed symptoms of colic for more than 24 hours and had not passed manure during this period. The abdomen was distended and the pony showed signs of pain. The baseline clinical values were: Temperature: 37.7 °C, heart rate: 70/min, respiratory rate: 30/min, Ht: 43%, TPP (g/dl): 8 g/dl. Before undergoing CT scanning and surgery, a laterolateral radiograph of the ventral abdomen was obtained, in which the presence of sand in the ventral colon was verified. Sand was also suspected to be present in the cecum (Additional file 2).

With the consent of the owner, we preoperatively performed a comprehensive abdominal and thoracic CT examination at the Department and Clinic of Equine Medicine of the University of Veterinary Medicine Budapest, with the animal in dorsal recumbency and a 70 cm FOV. The CT equipment and exposure settings were the same as in the first case. In this case we also performed Hounsfield Unit (HU) measurement (Additional file 3, 4) as an aid to identify different types of intestinal contents. CT images were analysed using a medical image visualization software (JiveX DICOM Viewer Version 5.2, Visus Health IT GmbH, Bochum, Germany) in multiplanar reconstruction, using an algorithm for soft tissue. The HU values from different intestinal segments (11 measurements) were recorded, as well as those from two parenchymal organs, the liver and the right kidney, were also recorded (Additional file 5). The HU values were measured by the same individual (PT). The region of interests (ROI)s were adjusted to the size of the area to be examined and therefore were of different size. They were designed not to include any other intestinal segments or organs.

After CT imaging of the thorax and abdomen and immediate comparative analysis of the images, the pony was maintained under general anaesthesia and underwent surgery immediately.

We used total intravenous anaesthesia during the CT scan. Premedication consisted of 1.1 mg/bwkg xylazine (CP-xylazine 20 mg/mL; Produlab Pharma BV, Raamsdonkveer, Netherlands) and 0,05 mg/bwkg butorphanol (Nalgosed 10 mg/mL; Bioveta a. s., Ivanovice na Hané, Czech Republic), followed by induction with 2,2 mg/bwkg ketamine (Narkamon 100 mg/mL; Bioveta a. s., Ivanovice na Hané, Czech Republic) and 0,05 mg/bwkg midazolam (Dormicum 5 mg/mL, Egis Pharmaceuticals PLC, Budapest, Hungary). The horse was intubated with a 16 mm cuffed silicone endotracheal tube (Kruuse, Denmark). After induction, the pony spontaneously positioned into right lateral recumbency and was transported to the CT room on a trolley, with left lateral recumbency. The CT examination was performed in dorsal recumbency. The subject was breathing spontaneously before and during the CT scan. The anaesthesiologist monitored the respiratory rate, which was low but within the normal range, and the images were obtained in two sections during the expiration phase. The cranial segment (thorax) was scanned first. After the CT examination the pony was transferred into the operating room and the anaesthesia was maintained with isoflurane (Isoflutek 1000 mg/g, Laboratorios Karizoo S.A., Spain) combined with infusion of lidocaine (LidoBel 20 mg/mL, Bela-pharm GmbH, Germany) in a continuous rate (50 mcg/bwkg/min). Assisted ventilation was also provided.

The procedure was planned with the help of the preoperative CT images. The surgical intervention included the evacuation of the impacted cecum and large colon through enterotomies of the cecum and the pelvic flexure (Additional file 6), which were expected preoperatively. The intestinal contents were examined macroscopically during surgery to identify gas, fluid, sand, and chewed contents and to estimate their proportions and amounts. There were no adverse events during the postoperative period, and the horse was discharged 10 days after surgery.

## Results

### Pony no. 1

**Fig. 1:**
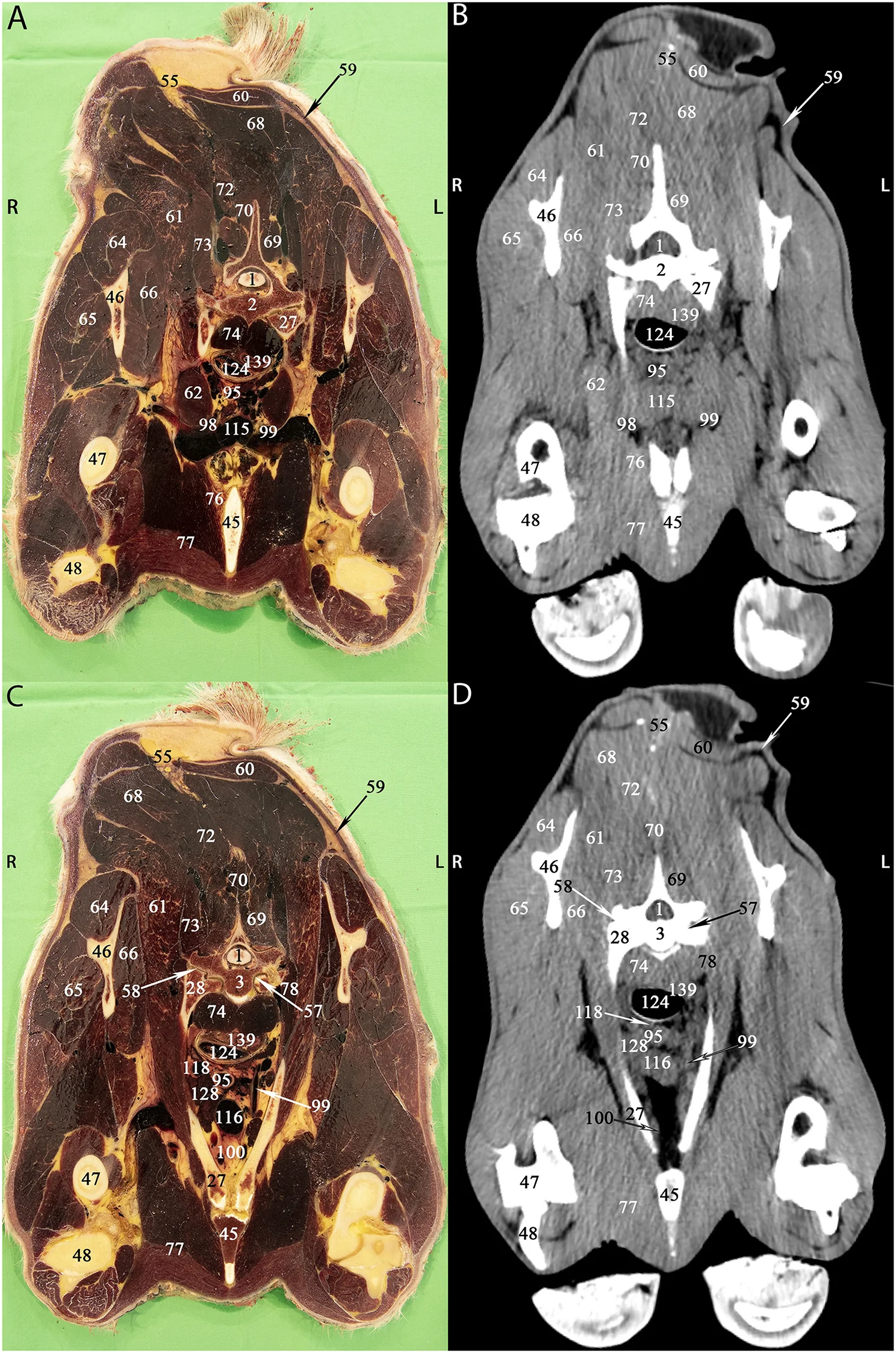
Comparative transverse image at the level of Th1–Th2. 1. *medulla spinalis* (spinal cord); 2. *vertebra Th1*; 3. *vertebra Th2*; 27. *costa 1*.; 28 *costa 2*.; 45. *sternum*; 46. *scapula*; 47. *humerus*; 48 *radius*; 55. *lig. nuchae*, 57. *art. capitis costae*, 58. *art. costotransversaria*, 59. *m. trapesius*; 60. *m. rhomboideus thoracis*; 61. *m. serratus ventralis*; 62. *m. scaleneus*; 64. *m. supraspinatus*; 65. *m. infraspinatus*; 66. *m. subscapularis; 68. m.* splenius; 69. *m.* multifidus; 70. *m. spinalis*; 72. *m. semispinalis*; 73. *m. longissimus cervicis*; 74. *m. longus colli*; 76. *m. subclavius*; 77. *mm. pectorales*; 78. *m. iliocostalis*; 95. *truncus bicaroticus*; 98. *a. subclavia dextra*; 99. *a. subclavia sinistra*; 100. *a. et v. thoracica interna*; 115. *truncus bijugularis*; 116. *v. cava cranialis*; 118. *v. costocercivalis*; 124. *trachea*; 128. *lnn. mediastinales craniales*; 139. *oesophagus*

**Fig. 2:**
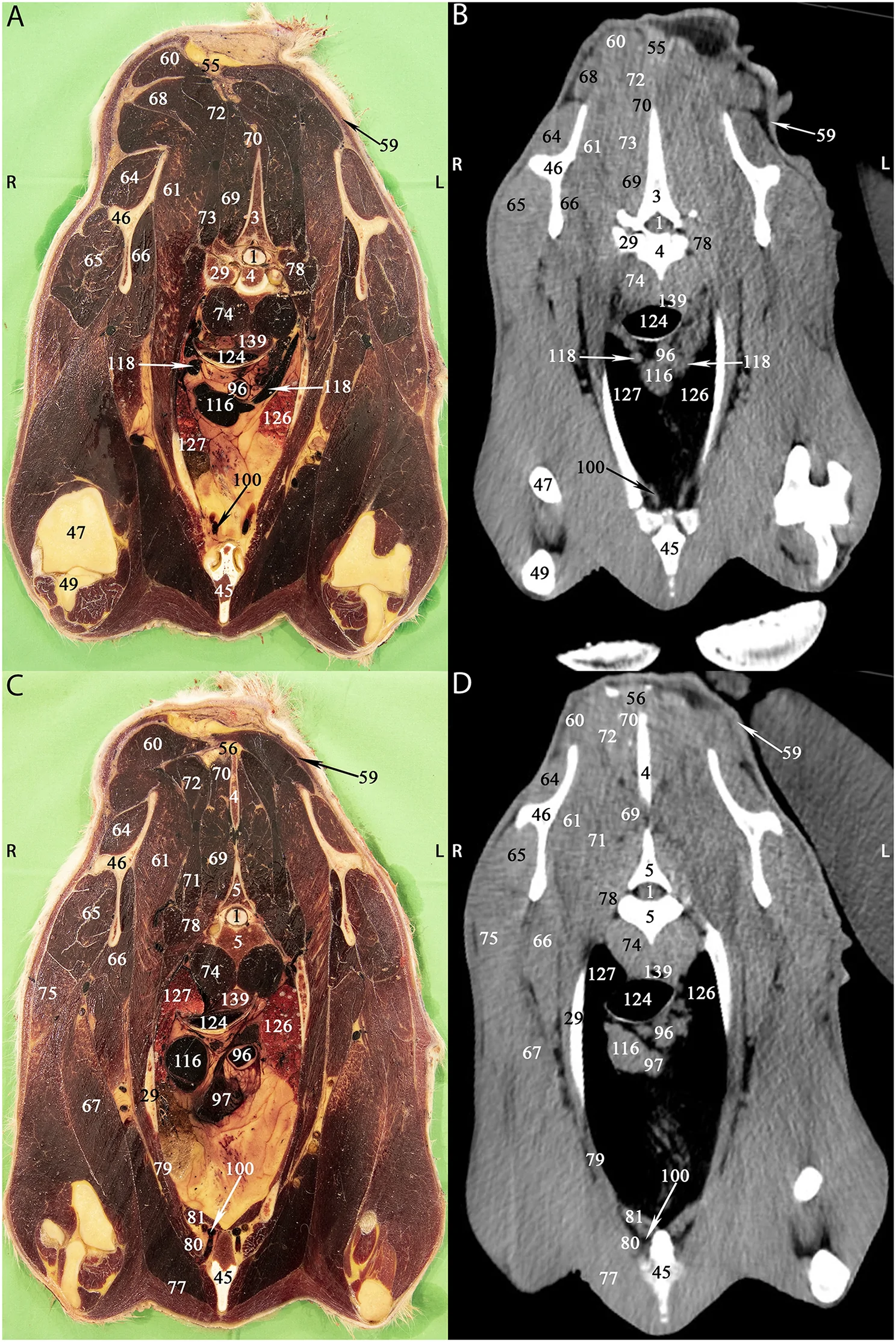
Comparative transverse image at the level of Th3–Th4. 1. *medulla spinalis* (spinal cord); 3. *vertebra Th2*; 4. *vertebra Th3*; 5. *vertebra Th4;* 29. *costa 3*.; 45. *sternum*; 46. *scapula*; 47. *humerus*; 49. *ulna*; 55. *lig. nuchae;* 56. *lig. supraspinale;* 59. *m. trapesius*; 60. *m. rhomboideus thoracis*; 61. *m. serratus ventralis*; 64. *m. supraspinatus*; 65. *m. infraspinatus*; 66. *m. subscapularis*; 67. *m. triceps brachii; 68. m.* splenius; 69. *m.* multifidus; 70. *m. spinalis*; 71. *m. longissimus dorsi*; 72. *m. semispinalis*; 74. *m. longus colli*; 75. *m. deltoideus*; 77. *mm. pectorales*; 78. *m. iliocostalis*; 79. *m. intercostales externi et interni*; 80. *mm. subcostales*; 81. *m. transversus thoracis*; 96. *truncus brachiocephalicus*; 97. *truncus pulmonalis*; 100. *a. et v. thoracica interna*; 116. *v. cava cranialis*; 118. *v. costocervicalis*; 124. *trachea*; 126. *pulmo sinister* (left lung); 127. *pulmo dexter* (right lung); 139. *oesophagus*

**Fig. 3:**
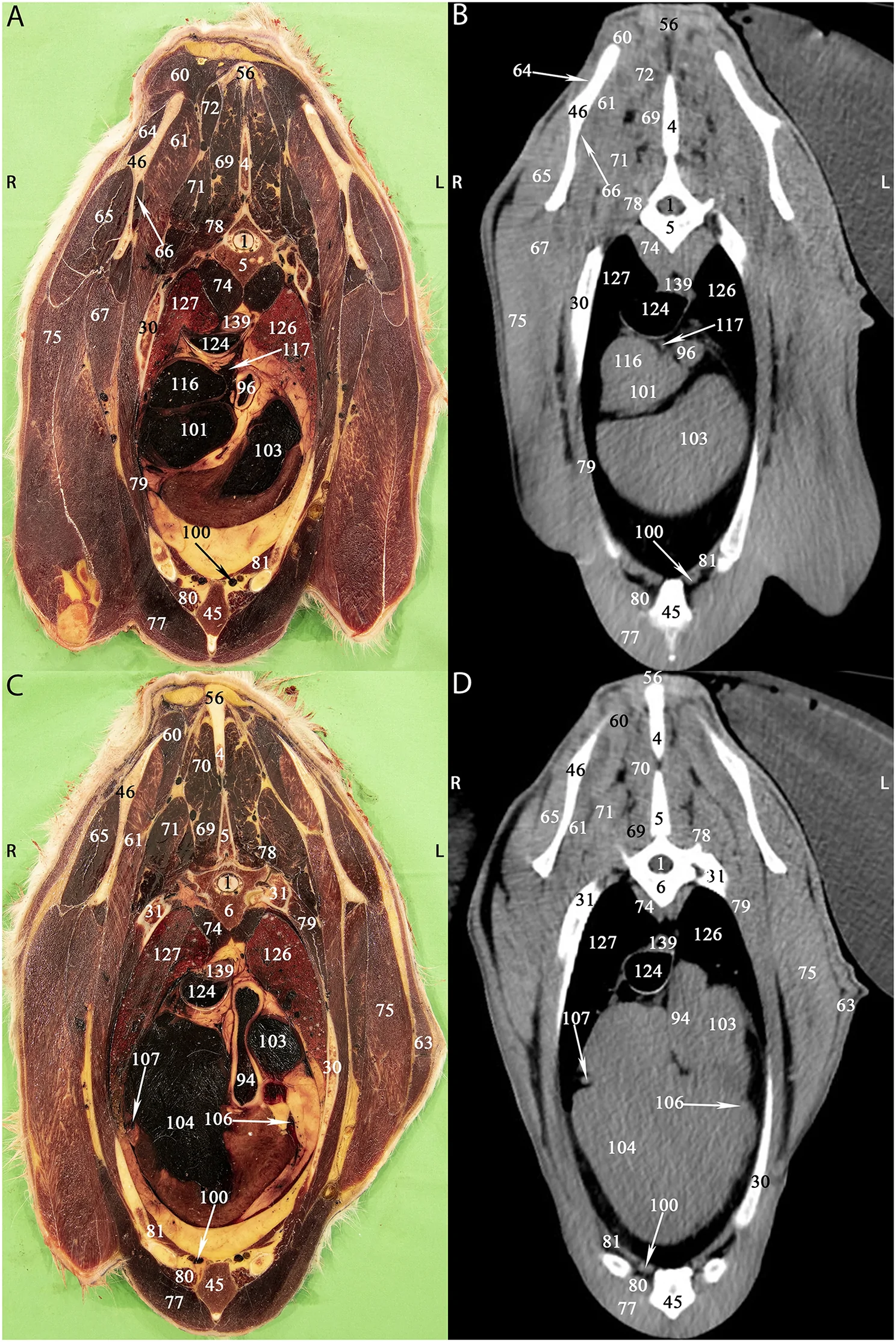
Comparative transverse image at the level of Th4–Th5. 1. *medulla spinalis* (spinal cord); 4. *vertebra Th3*; 5. *vertebra Th4;* 6. *vertebra Th5;* 30. *costa 4*.; 31. *costa 5.*; 45. *sternum*; 46. *scapula*; 56. *lig. supraspinale*; 60. *m. rhomboideus thoracis*; 61. *m. serratus ventralis*; 63. *m. latissimus dorsi* 64. *m. supraspinatus* 65. *m. infraspinatus*; 66. *m. subscapularis*; 67. *m. triceps brachii*; 69. *m.* multifidus; 70. *m. spinalis*; 71. *m. longissimus dorsi*; 72. *m. semispinalis*; 74. *m. longus colli*; 75. *m. deltoideus* 77. *mm. pectorales*; 78. *m. iliocostalis*; 79. *m. intercostales externi et interni*; 80. *mm. subcostales*; 81. *m. transversus thoracis*; 94. *aorta*; 96. *truncus brachiocephalicus*; 100. *a. et v. thoracica interna*; 101. *auricula dextra*; 103. *atrium sinistrum*; 104. *ventriculus dexter*; 106. *a. coronaria sinistra*; 107. *a. coronaria dextra*; 116. *v. cava cranialis*; 117. *v. azygos dextra*; 124. *trachea*; 126. *pulmo sinister* (left lung); 127. *pulmo dexter* (right lung); 139. *oesophagus*

**Fig. 4:**
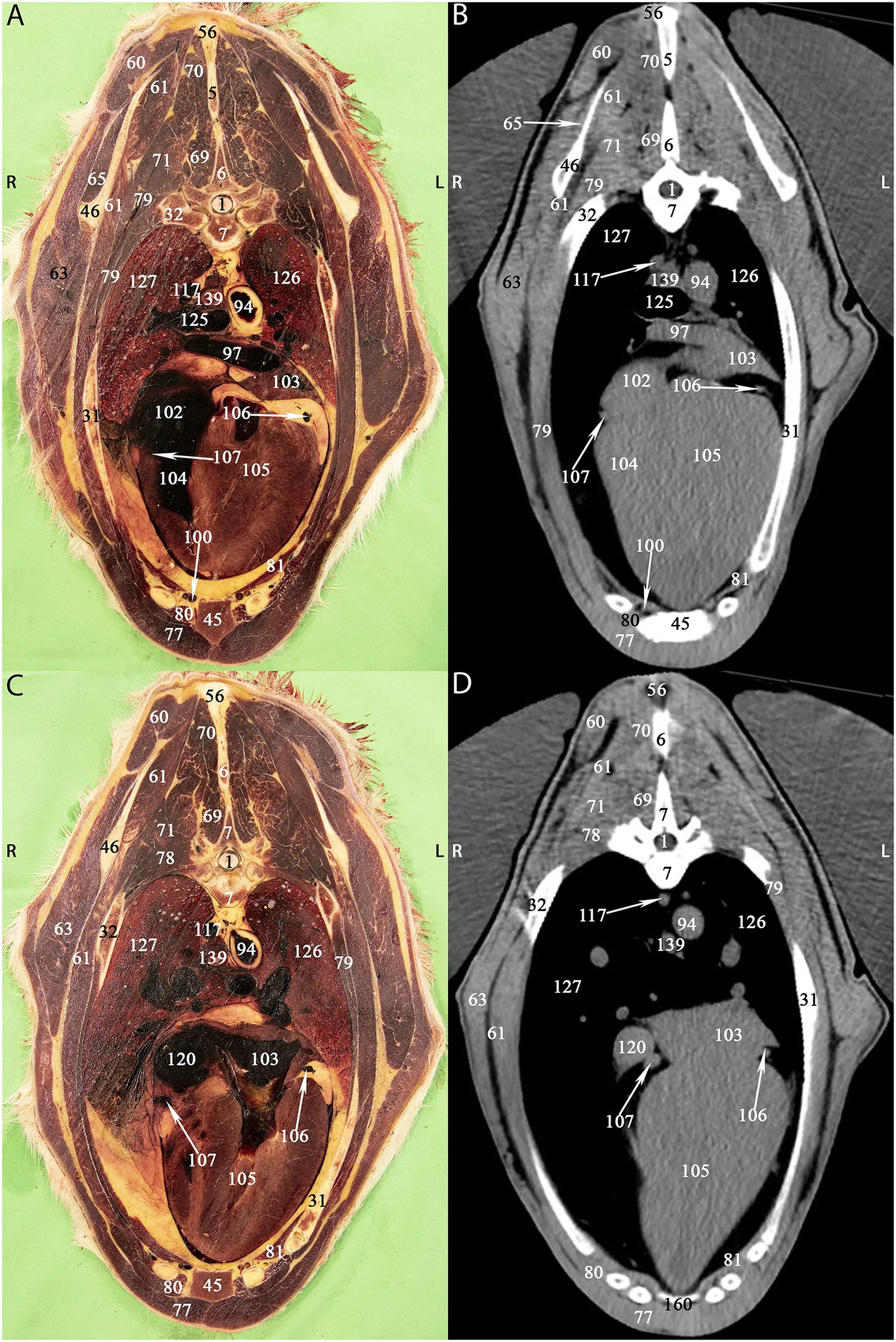
Comparative transverse image at the level of Th5–Th6. 1. *medulla spinalis* (spinal cord); 5. *vertebra Th4;* 6. *vertebra Th5;* 7. *vertebra Th6*; 31. *costa 5.*; 32. *costa 6*.;45. *sternum*; 46. *scapula*; 56. *lig. supraspinale;* 60. *m. rhomboideus thoracis*; 61. *m. serratus ventralis*; 63. *m. latissimus dorsi*; 65. *m. infraspinatus*; 69. *m.* multifidus; 70. *m. spinalis*; 71. *m. longissimus dorsi*; 77. *mm. pectorales*; 78. *m. iliocostalis*; 79. *m. intercostales externi et interni*; 80. *mm. subcostales*; 81. *m. transversus thoracis*; 94. *aorta*; 97. *truncus pulmonaris* 100. *a. et v. thoracica interna*; 102. *atrium dextrum*; 103. *atrium sinistrum*; 104. *ventriculus dexter*; 105. *ventriculus sinister*; 106. *a. coronaria sinistra*; 107. *a. coronaria dextra*; 117. *v. azygos dextra*; 120. *v. cava caudalis* 125. *bifurcatio tracheae*; 126. *pulmo sinister* (left lung); 127. *pulmo dexter* (right lung); 139. *oesophagus* 160. *proc. Xiphoideus*

**Fig. 5:**
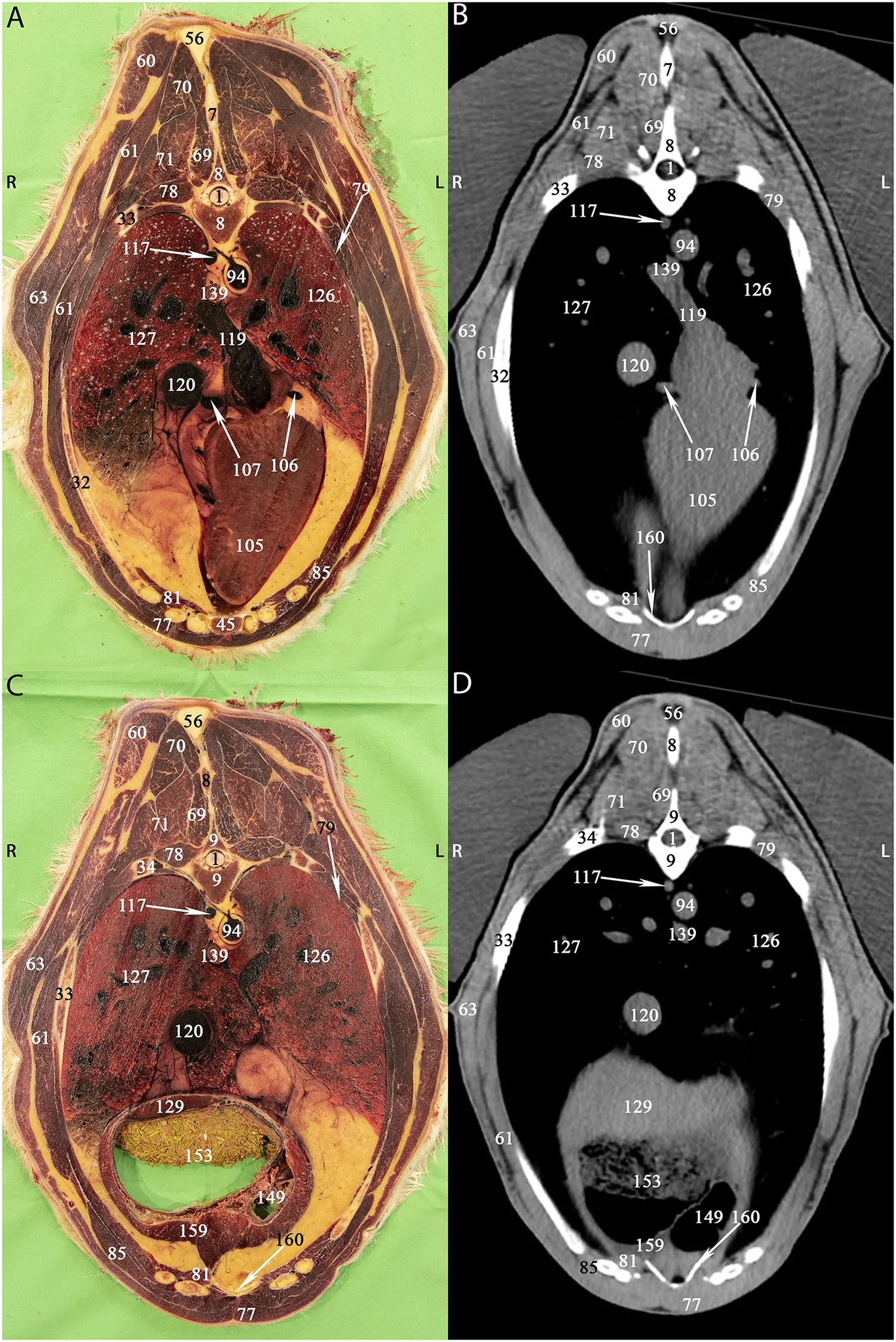
Comparative transverse image at the level of Th7–Th8. 1. *medulla spinalis* (spinal cord); 7. *vertebra Th6;* 8. *vertebra Th7;* 9. *vertebra Th8*; 32. *costa 6.*; 33. *costa 7*.; 34. *costa 8.*; 45. *sternum*; 56. *lig. supraspinale;* 60. *m. rhomboideus thoracis*; 61. *m. serratus ventralis*; 63. *m. latissimus dorsi*; 69. *m.* multifidus; 70. *m. spinalis*; 71. *m. longissimus dorsi*; 77. *mm. pectorales*; 78. *m. iliocostalis*; 79. *m. intercostales externi et interni*; 81. *m. transversus thoracis*; 85. *m. rectus abdominis*; 94. *aorta*; 105. *ventriculus sinister*; 106. *a. coronaria sinistra*; 107. *a. coronaria dextra*; 117. *v. azygos dextra*; 119. *vv. pulmonales* 120. *v. cava caudalis*; 126. *pulmo sinister* (left lung); 127. *pulmo dexter* (right lung); 129. *hepar* (liver); 139. *oesophagus*; 149. *flexura sternalis* (sternal flexure); 153. *flexura diaphragmatica* (diaphragmatic flexure); 159. *diaphragma*; 160. *proc. Xiphoideus*

**Fig. 6:**
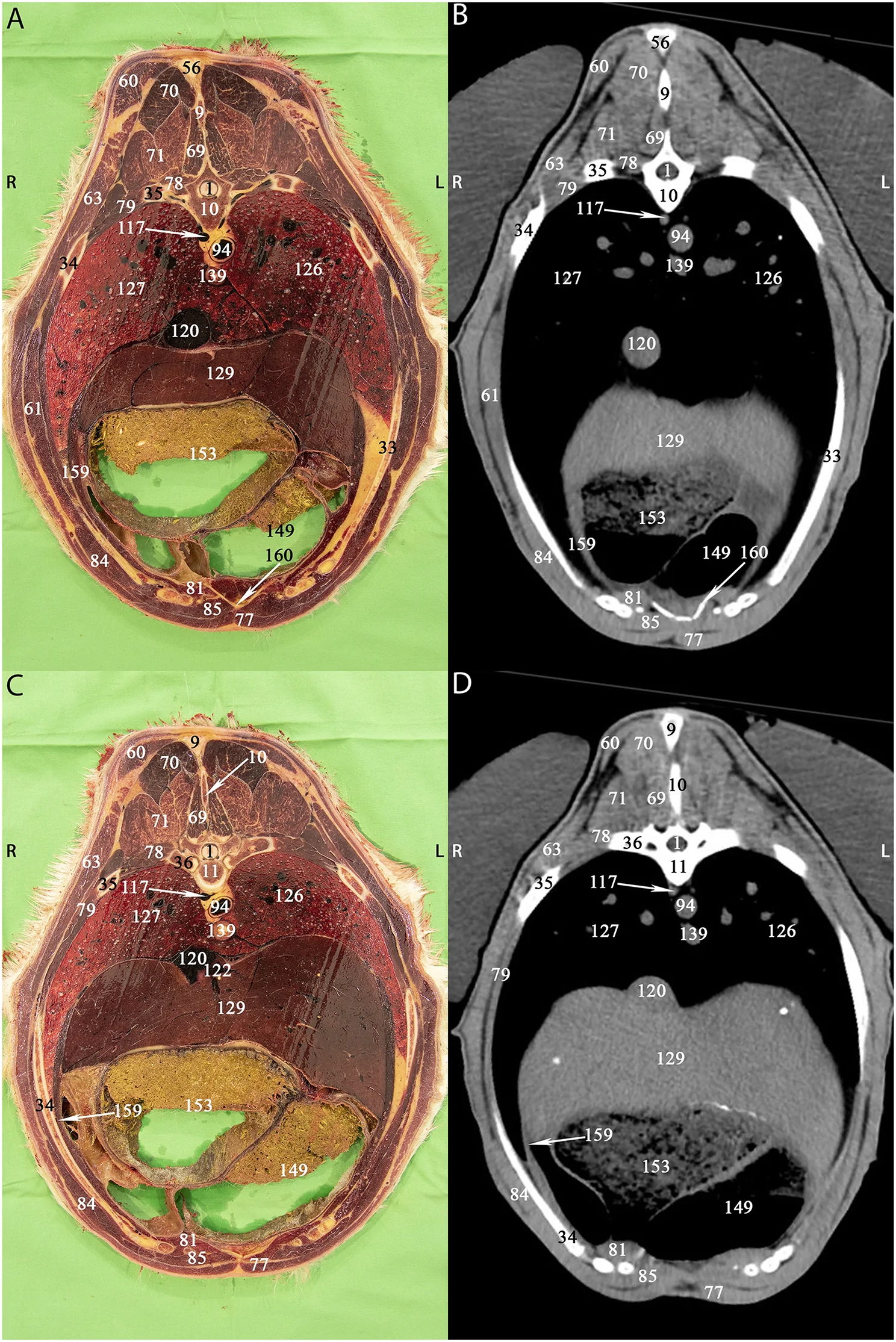
Comparative transverse image at the level of Th9–Th10. 1. *medulla spinalis* (spinal cord); 9. *vertebra Th8*; 10. *vertebra Th9*; 11. *vertebra Th10*; 33. *costa 7.;* 34. *costa 8.*; 35. *costa 9*.; 36. *costa 10.*; 56. *lig. supraspinale;* 60. *m. rhomboideus thoracis*; 61. *m. serratus ventralis*; 63. *m. latissimus dorsi*; 69. *m.* multifidus; 70. *m. spinalis*; 71. *m. longissimus dorsi*; 77. *mm. pectorales*; 78. *m. iliocostalis*; 79. *m. intercostales externi et interni*; 81. *m. transversus thoracis*; 84. *m. obliquus externus abdominis*; 85. *m. rectus abdominis*; 94. *aorta*; 117. *v. azygos dextra*; 120. *v. cava caudalis*; 122. *vv. hepaticae* 126. *pulmo sinister* (left lung); 127. *pulmo dexter* (right lung); 129. *hepar* (liver); 139. *oesophagus*; 149. *flexura sternalis* (sternal flexure); 153. *flexura diaphragmatica* (diaphragmatic flexure); 159. *diaphragma*; 160. *proc. Xiphoideus*

**Fig. 7:**
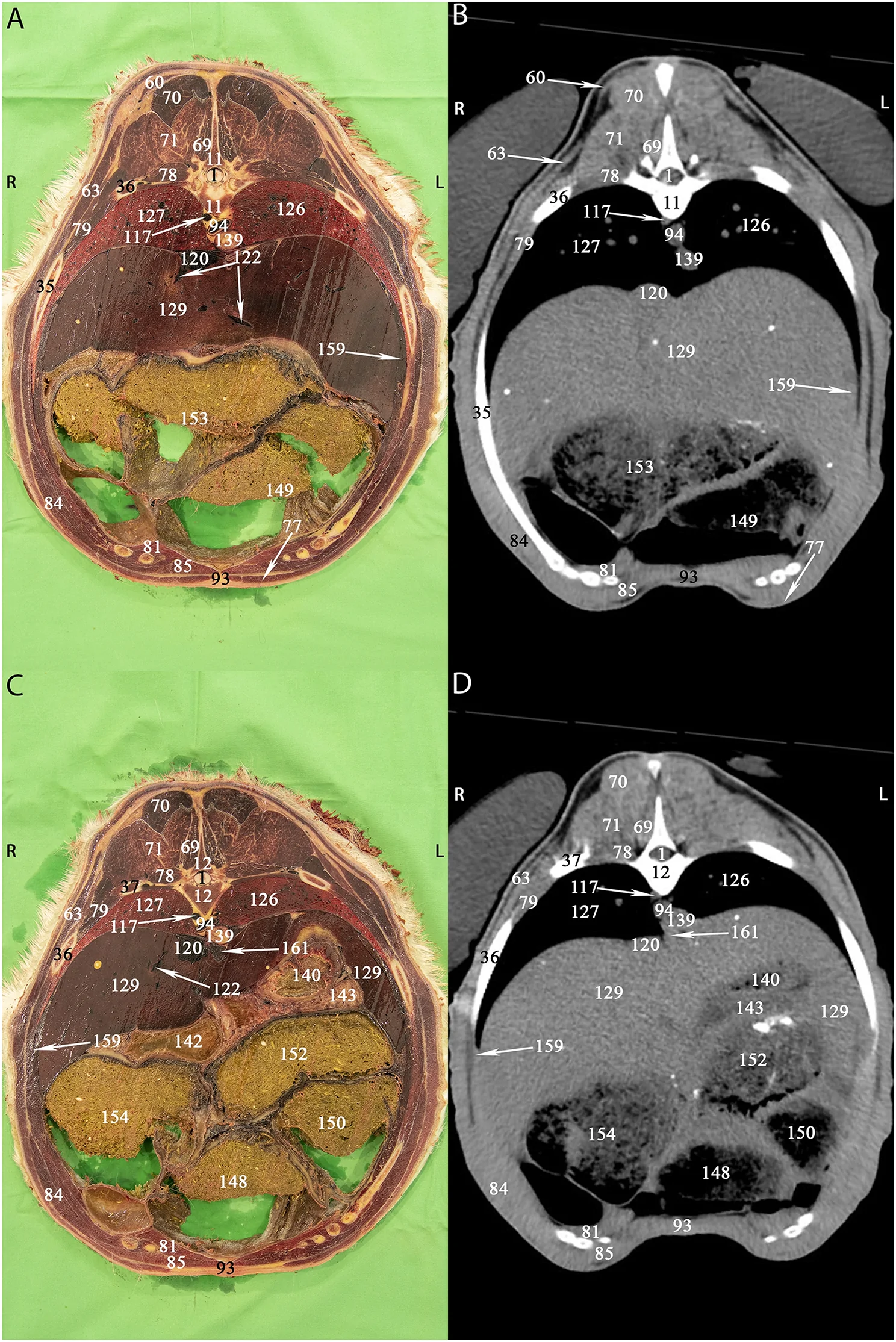
Comparative transverse image at the level of Th10–Th11. 1. *medulla spinalis* (spinal cord); 11. *vertebra Th10*; 12. *vertebra Th11*; 35. *costa 9*.; 36. *costa 10.*; 37. *costa 11.*; 60. *m. rhomboideus thoracis*; 63. *m. latissimus dorsi*; 69. *m.* multifidus; 70. *m. spinalis*; 71. *m. longissimus dorsi*; 77. *mm. pectorales*; 78. *m. iliocostalis*; 79. *m. intercostales externi et interni*; 81. *m. transversus thoracis*; 84. *m. obliquus externus abdominis*; 85. *m. rectus abdominis*; 93. *linea alba*; 94. *aorta*; 117. *v. azygos dextra*; 120. *v. cava caudalis*; 122. *vv. hepaticae*; 126. *pulmo sinister* (left lung); 127. *pulmo dexter* (right lung); 129. *hepar* (liver); 139. *oesophagus*; 140. *ventriculus* (stomach); 142. *duodenum*; 143. *jejunum*; 148. *colon ventrale dextrum* (right ventral colon); 149. *flexura sternalis* (sternal flexure); 150. *colon ventrale sinistrum* (left ventral colon); 152. *colon dorsale sinistrum* (right dorsal colon); 153. *flexura diaphragmatica* (diaphragmatic flexure); 154. *colon dorsale dextrum* (right dorsal colon); 159. *diaphragma*; 161. *diaphragma pars lumbalis*

**Fig. 8:**
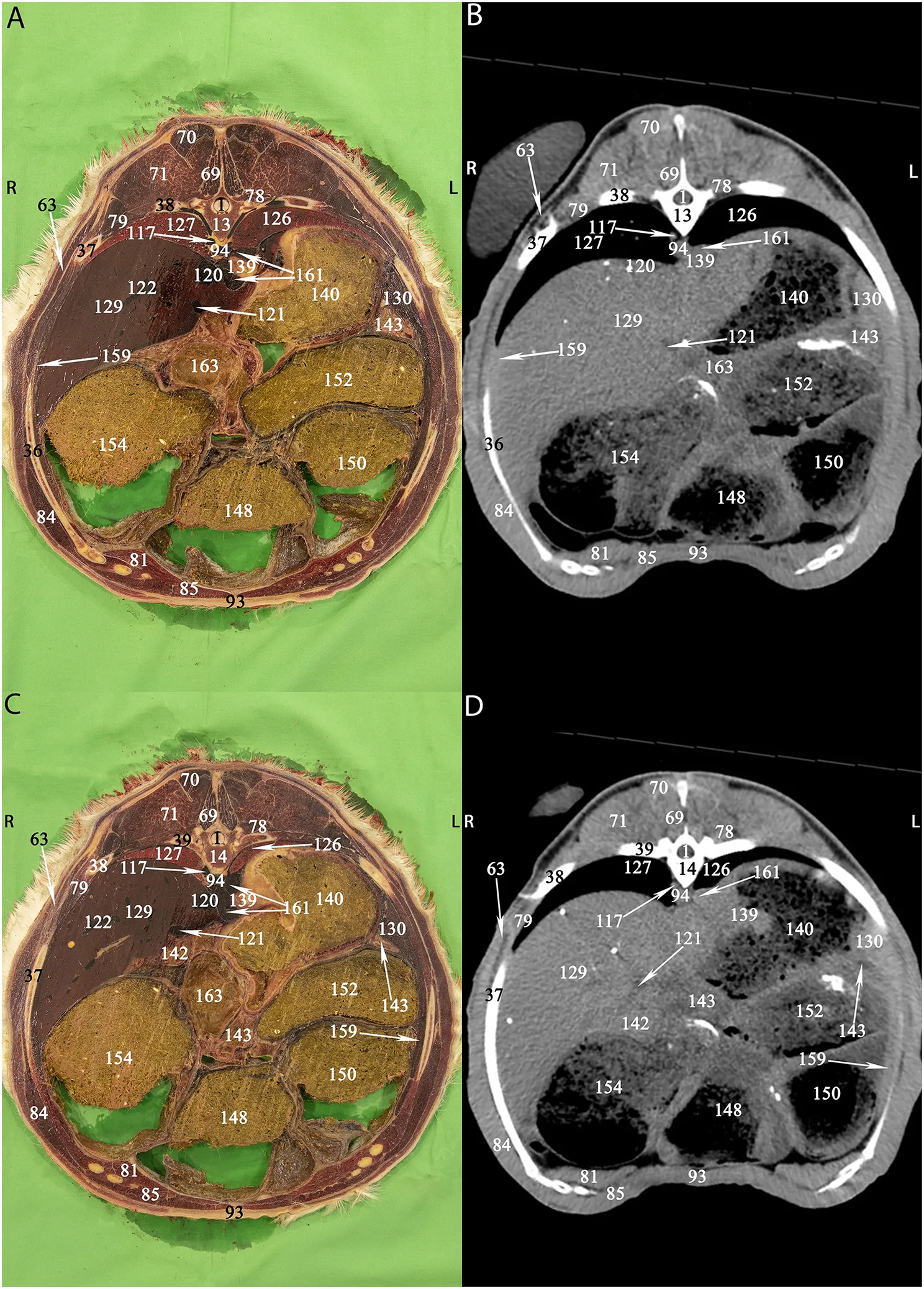
Comparative transverse image at the level of Th12–Th13. 1. *medulla spinalis* (spinal cord); 13. *vertebra Th12*; 14. *vertebra Th13*; 36. *costa 10.*; 37. *costa 11*.; 38. *costa 12.*; 39. *costa 12.;* 63. *m. latissimus dorsi*; 69. *m.* multifidus; 70. *m. spinalis*; 71. *m. longissimus dorsi*; 78. *m. iliocostalis*; 79. *m. intercostales externi et interni*; 81. *m. transversus thoracis*; 84. *m. obliquus externus abdominis*; 85. *m. rectus abdominis*; 93. *linea alba*; 94. *aorta*; 117. *v. azygos dextra*; 120. *v. cava caudalis*; 121. *v. portae*; 122. *vv. hepaticae;* 126. *pulmo sinister* (left lung); 127. *pulmo dexter* (right lung); 129. *hepar* (liver); 130. *lien* (spleen); 139. *oesophagus* 140. *ventriculus* (stomach); 142. *duodenum*; 143. *jejunum*; 148. *colon ventrale dextrum* (right ventral colon); 150. *colon ventrale sinistrum* (left ventral colon); 152. *colon dorsale sinistrum* (left dorsal colon); 154. *colon dorsale dextrum* (right dorsal colon); 159. *diaphragma*; 161. *diaphragma pars lumbalis*; 163. *ampulla duodeni*

**Fig. 9:**
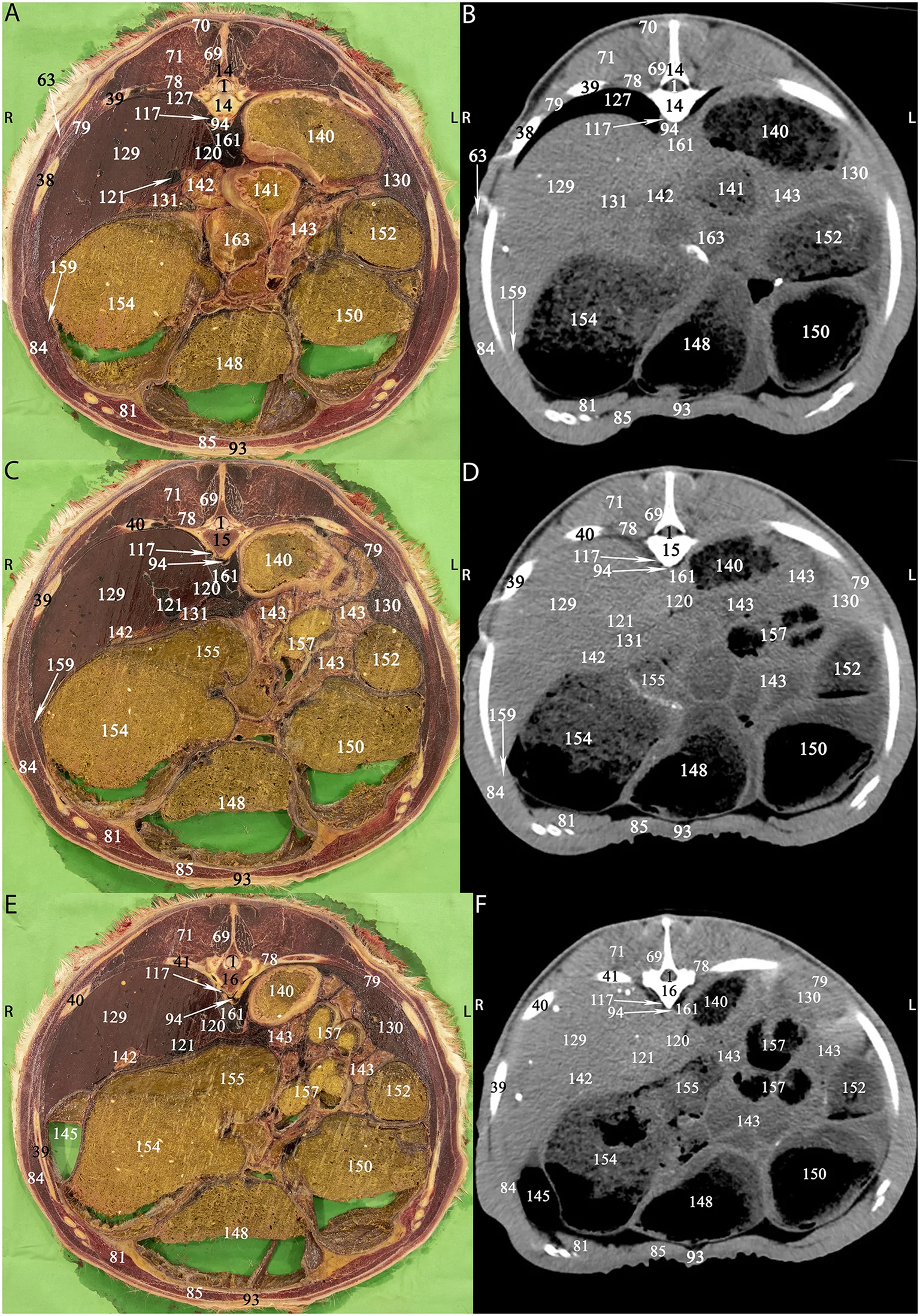
Comparative transverse image at the level of Th13–Th15. 1. *medulla spinalis* (spinal cord); 14. *vertebra Th13*; 15. *vertebra Th14*; 16. *vertebra Th15*; 38. *costa 12.*; 39. *costa 13.;* 40. *costa 14.*; 41. *costa 15.*; 63. *m. latissimus dorsi*; 69. *m.* multifidus; 70. *m. spinalis*; 71. *m. longissimus dorsi*; 78. *m. iliocostalis*; 79. *m. intercostales externi et interni*; 81. *m. transversus thoracis*; 84. *m. obliquus externus abdominis*; 85. *m. rectus abdominis*; 93. *linea alba*; 94. *aorta*; 117. *v. azygos dextra*; 120. *v. cava caudalis*; 121. *v. portae*; 127. *pulmo dexter* (right lung); 129. *hepar* (liver); 130. *lien* (spleen); 131. *pancreas*; 140. *ventriculus* (stomach); 141. *pylorus*; 142. *duodenum*; 143. *jejunum*; 145. *caput ceci* (head of the cecum); 148. *colon ventrale dextrum* (right ventral colon); 150. *colon ventrale sinistrum* (left ventral colon); 152. *colon dorsale sinistrum* (left dorsal colon); 154. *colon dorsale dextrum* (right dorsal colon); 155. *ampulla coli;* 157. *colon tenue* (small colon); 159. *diaphragma*; 161. *diaphragma pars lumbalis*

**Fig. 10:**
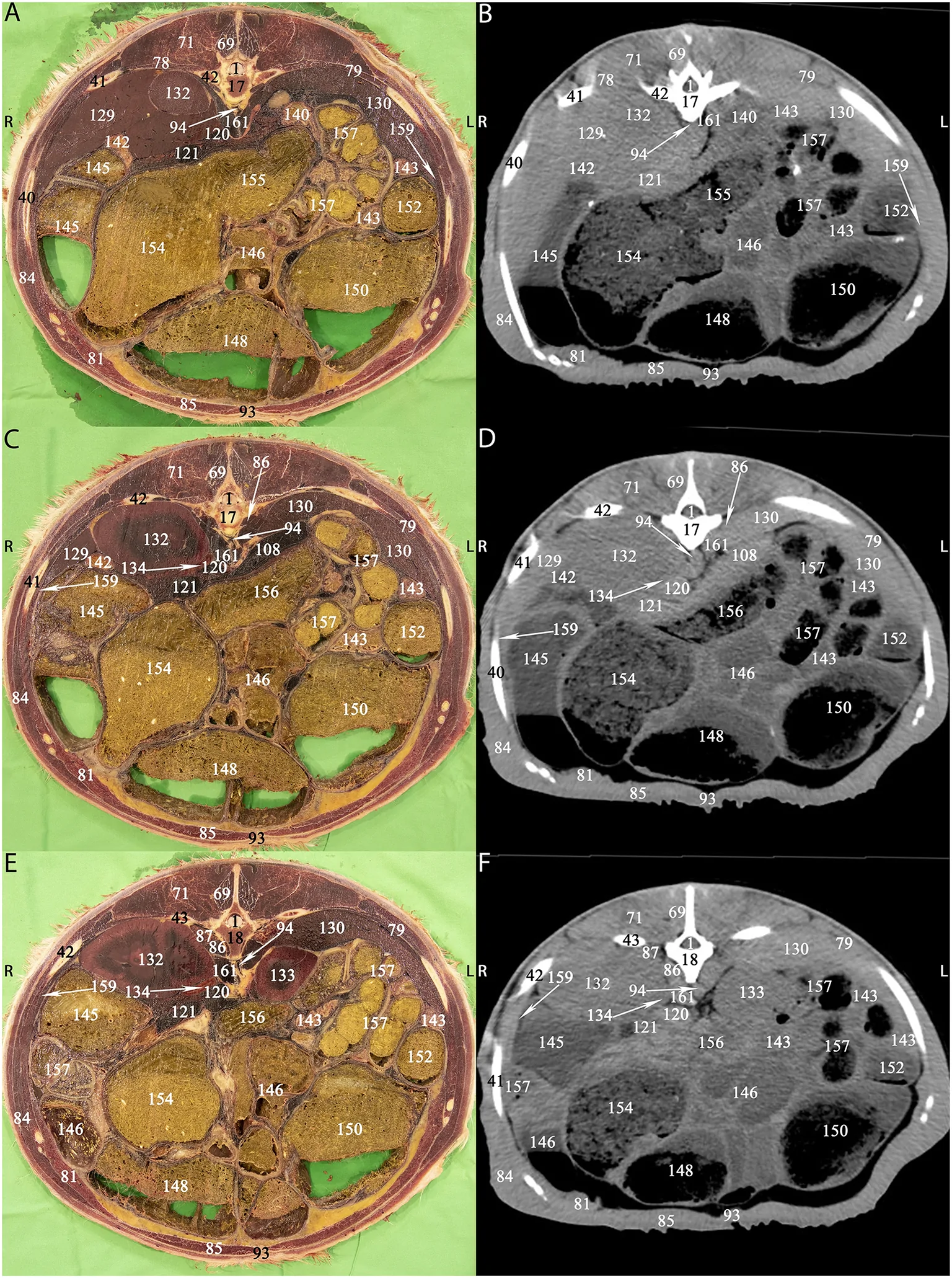
Comparative transverse image at the level of Th16–Th17. 1. *medulla spinalis* (spinal cord); 17. *vertebra Th16*; 18. *vertebra Th17*; 40. *costa 14.*; 41. *costa 15.*; 42. *costa 16.*; 43. *costa 17.*; 69. *m.* multifidus; 71. *m. longissimus dorsi*; 79. *m. intercostales externi et interni*; 81. *m. transversus thoracis*; 84. *m. obliquus externus abdominis*; 85. *m. rectus abdominis*; 86. *m. psoas minor*; 87. *m. psoas major*; 93. *linea alba*; 94. *aorta*; 108. *a. lienalis*; 120. *v. cava caudalis*; 121. *v. portae*; 129. *hepar* (liver); 130. *lien* (spleen); 132. *ren dextra* (right kidney); 133. *ren sinistra* (left kidney); 134. *glandula adrenalis dextra*; 140. *ventriculus* (stomach); 142. *duodenum*; 143. *jejunum*; 145. *caput ceci* (head of the cecum); 146. *corpus ceci* (body of the cecum); 148. *colon ventrale dextrum* (right ventral colon); 150. *colon ventrale sinistrum* (left ventral colon); 152. *colon dorsale sinistrum* (left dorsal colon); 154. *colon dorsale dextrum* (right dorsal colon); 155. *ampulla coli*; 156. *colon transversum* (transverse colon); 157. *colon tenue* (small colon); 159. *diaphragma*; 161. *diaphragma pars lumbalis*

**Fig. 11:**
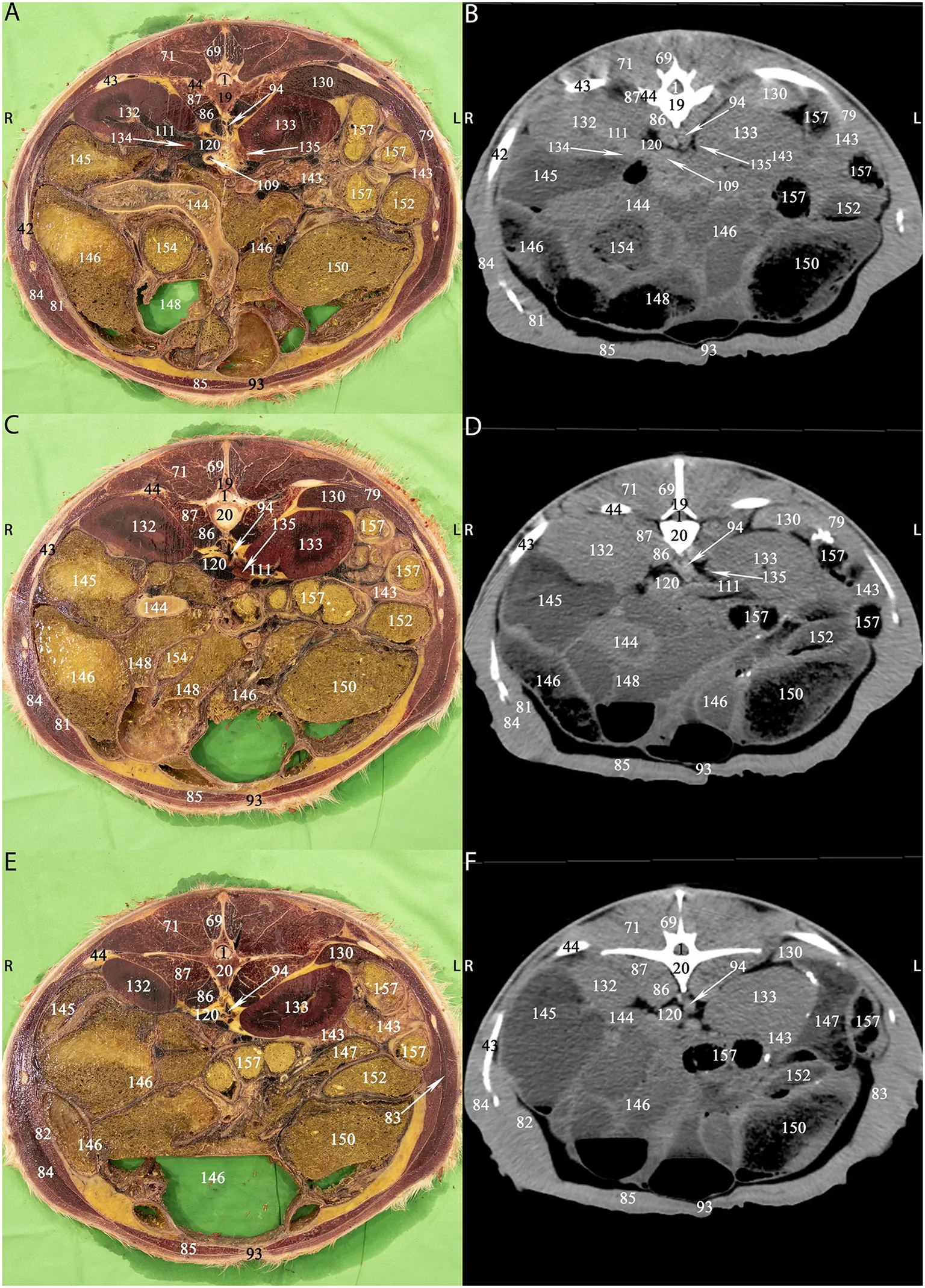
Comparative transverse image at the level of Th18–L1. 1. *medulla spinalis* (spinal cord); 19. *vertebra Th18*; 20. *vertebra L1*; 42. *costa 16*.; 43. *costa 17.*; 44. *costa 18.*; 69. *m.* multifidus; 71. *m. longissimus dorsi*; 79. *m. intercostales externi et interni*; 81. *m. transversus thoracis*; 82. *m. transversus abdominis*; 83. *m. obliquus internus abdominis* 84. *m. obliquus externus abdominis*; 85. *m. rectus abdominis*; 86. *m. psoas minor*; 87. *m. psoas major*; 93. *linea alba*; 94. *aorta*; 109. *a. mesenterica cranialis*; 111. *a. et v. renalis*; 120. *v. cava caudalis*; 130. *lien* (spleen); 132. *ren dextra* (right kidney); 133. *ren sinistra* (left kidney); 134. *glandula adrenalis dextra*; 135. *glandula adrenalis sinistra*; 143. *jejunum*; 144. *ileum*; 145. *caput ceci* (head of the cecum); 146. *corpus ceci* (body of the cecum); 147. *apex ceci* (apex of the cecum); 148. *colon ventrale dextrum* (right ventral colon); 150. *colon ventrale sinistrum* (left ventral colon); 152. *colon dorsale sinistrum* (left dorsal colon); 154. *colon dorsale dextrum (*right dorsal colon); 157. *colon tenue* (small colon)

**Fig. 12:**
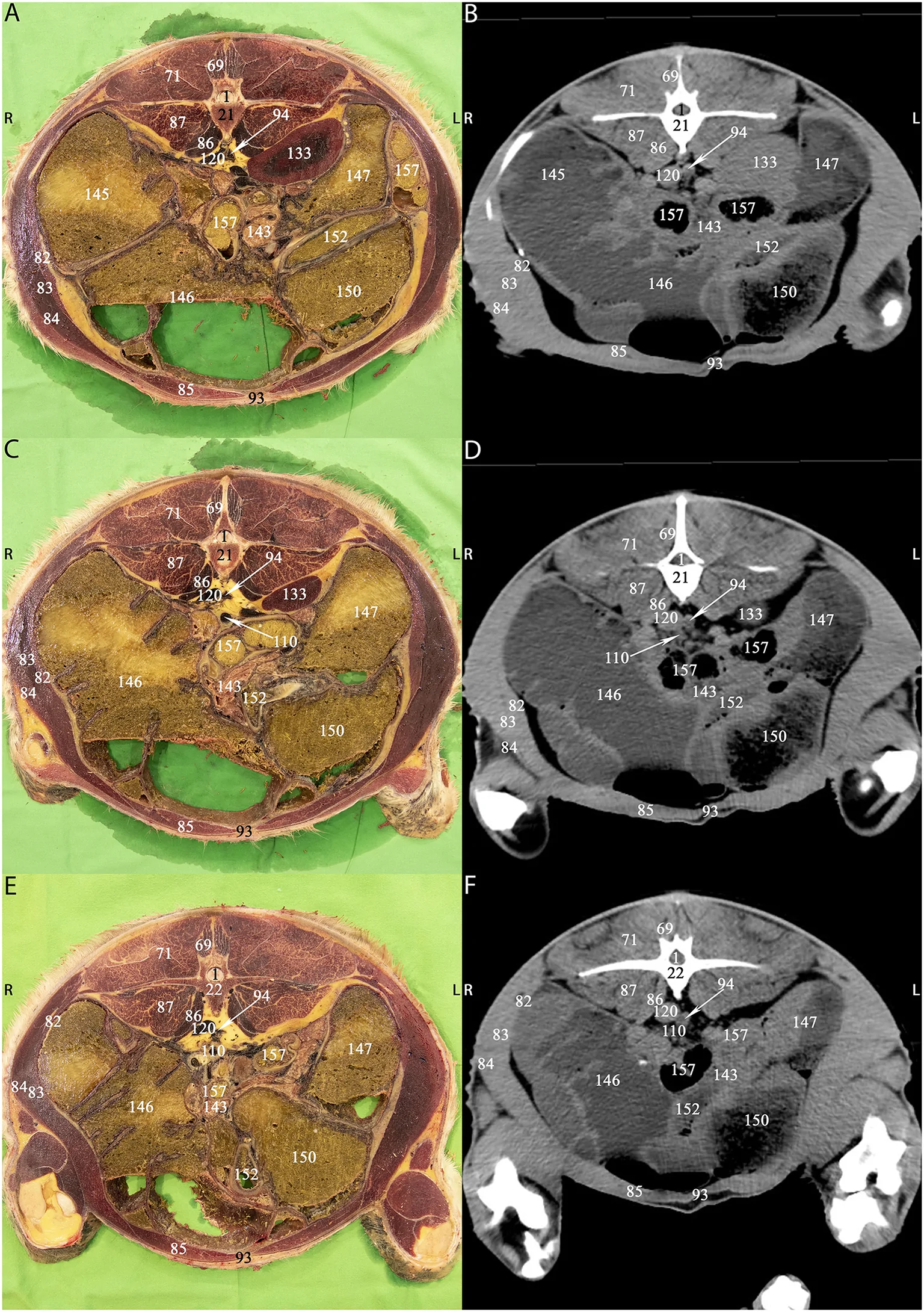
Comparative transverse image at the level of L2–L3. 1. *medulla spinalis* (spinal cord); 21. *vertebra L2;* 22. *vertebra L3;* 69. *m. multifidus;* 71. *m. longissimus dorsi;* 82. *m. transversus abdominis;* 83. *m. obliquus internus abdominis* 84. *m. obliquus externus abdominis;* 85. *m. rectus abdominis;* 86. *m. psoas minor;* 87. *m. psoas major;* 93. *linea alba;* 94. *aorta;* 110. *a. mesenterica caudalis;* 120. *v. cava caudalis;* 133. *ren sinistra* (left kidney); 143. *jejunum;* 145. *caput ceci* (head of the cecum); 146. *corpus ceci* (body of the cecum); 147. *apex ceci* (apex of the cecum); 150. *colon ventrale sinistrum* (left ventral colon); 152. *colon dorsale sinistrum* (left dorsal colon); 157. *colon tenue* (small colon)

**Fig. 13:**
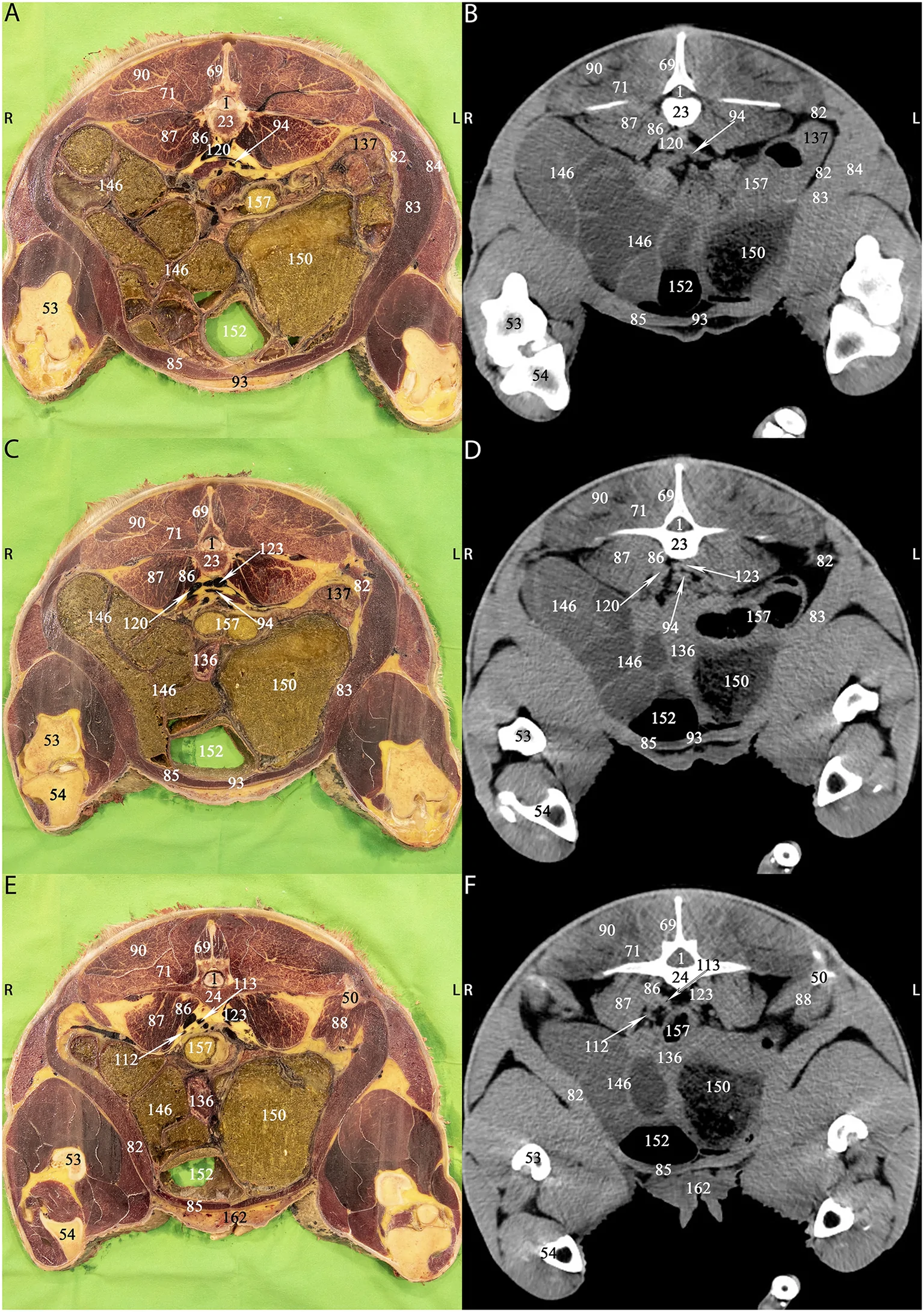
Comparative transverse image at the level of L4–L5. 1. *medulla spinalis* (spinal cord); 23. *vertebra L4;* 24. *vertebra L5;* 50. *os ilium;* 53. *femur;* 54. *tibia;* 69. *m. multifidus;* 71. *m. longissimus dorsi;* 82. *m. transversus abdominis;* 83. *m. obliquus internus abdominis* 84. *m. obliquus externus abdominis;* 85. *m. rectus abdominis;* 86. *m. psoas minor;* 87. *m. psoas major;* 88. *m. iliacus;* 90. *m. gluteus medius;* 93. *linea alba;* 94. *aorta;* 112. *a. iliaca externa;* 113. *a. iliaca interna;* 120. *v. cava caudalis;* 123. *v. iliaca communis;* 136. *uterus;* 137. *ovarium sinistrum* (left ovary); 146. *corpus ceci* (body of the cecum); 150. *colon ventrale sinistrum* (left ventral colon); 152. *colon dorsale sinistrum* (left dorsal colon); 157. *colon tenuei* (small colon); 162. *glandula mammaria*

**Fig. 14:**
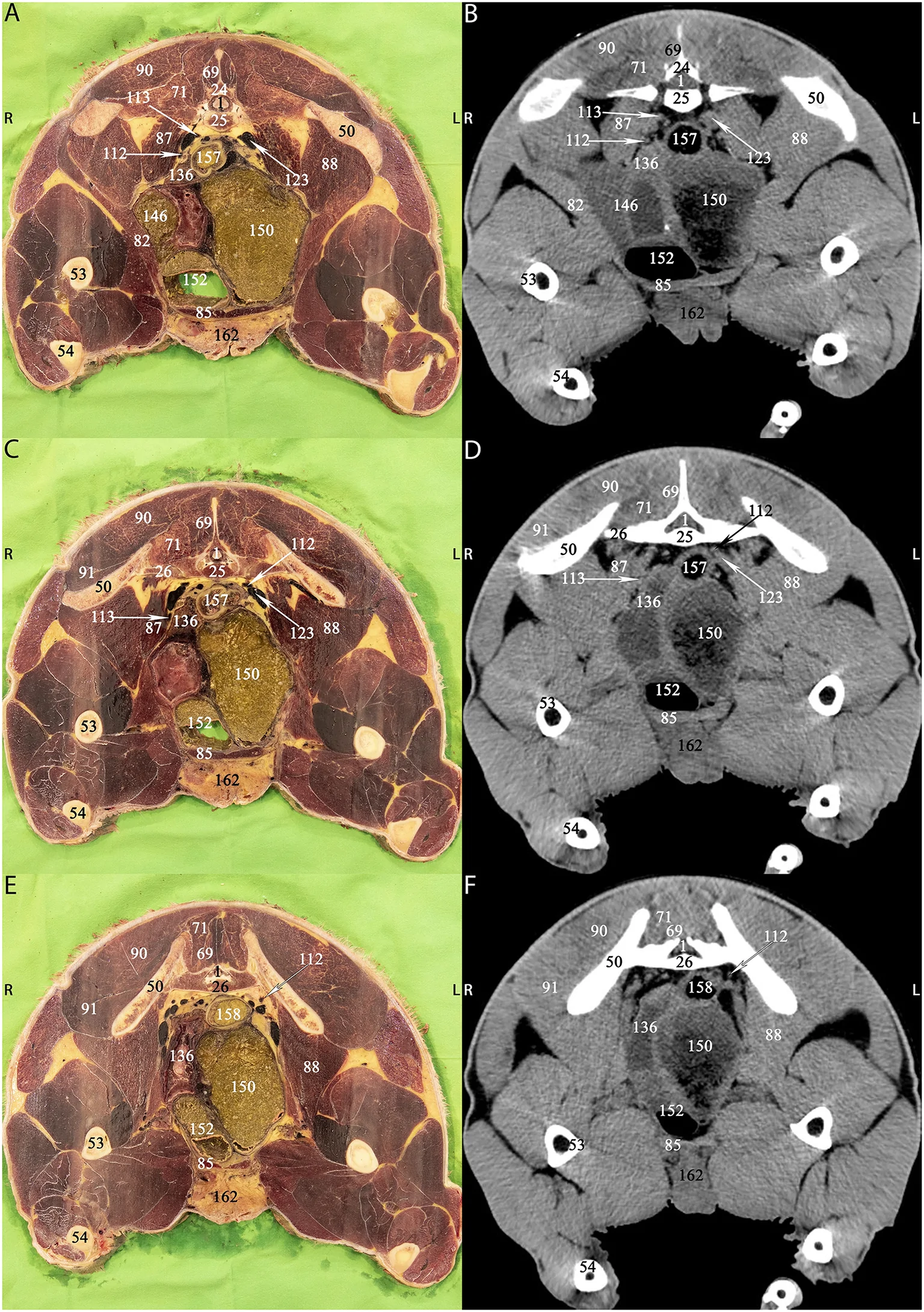
Comparative transverse image at the level of L6–S1. 1. *medulla* spinalis (spinal cord); 25. *vertebra L6;* 26. *os sacrum;* 50. *os ilium;* 53. *femur;* 54. *tibia;* 69. *m. multifidus;* 71. *m. longissimus dorsi;* 85. *m. rectus abdominis;* 87. *m. psoas major;* 88. *m. iliacus;* 90. *m. gluteus medius;* 91. *m. gluteus profundus;* 112. *a. iliaca externa* 113. *a. iliaca interna;* 123. *v. iliaca communis;* 136. *uterus;* 146. *corpus ceci* (body of the cecum); 150. *colon ventrale sinistrum* (left ventral colon); 152. *colon dorsale sinistrum* (left dorsal colon); 157. *colon tenuei* (small colon); 158. *rectum;* 162. *glandula mammaria*

**Fig. 15:**
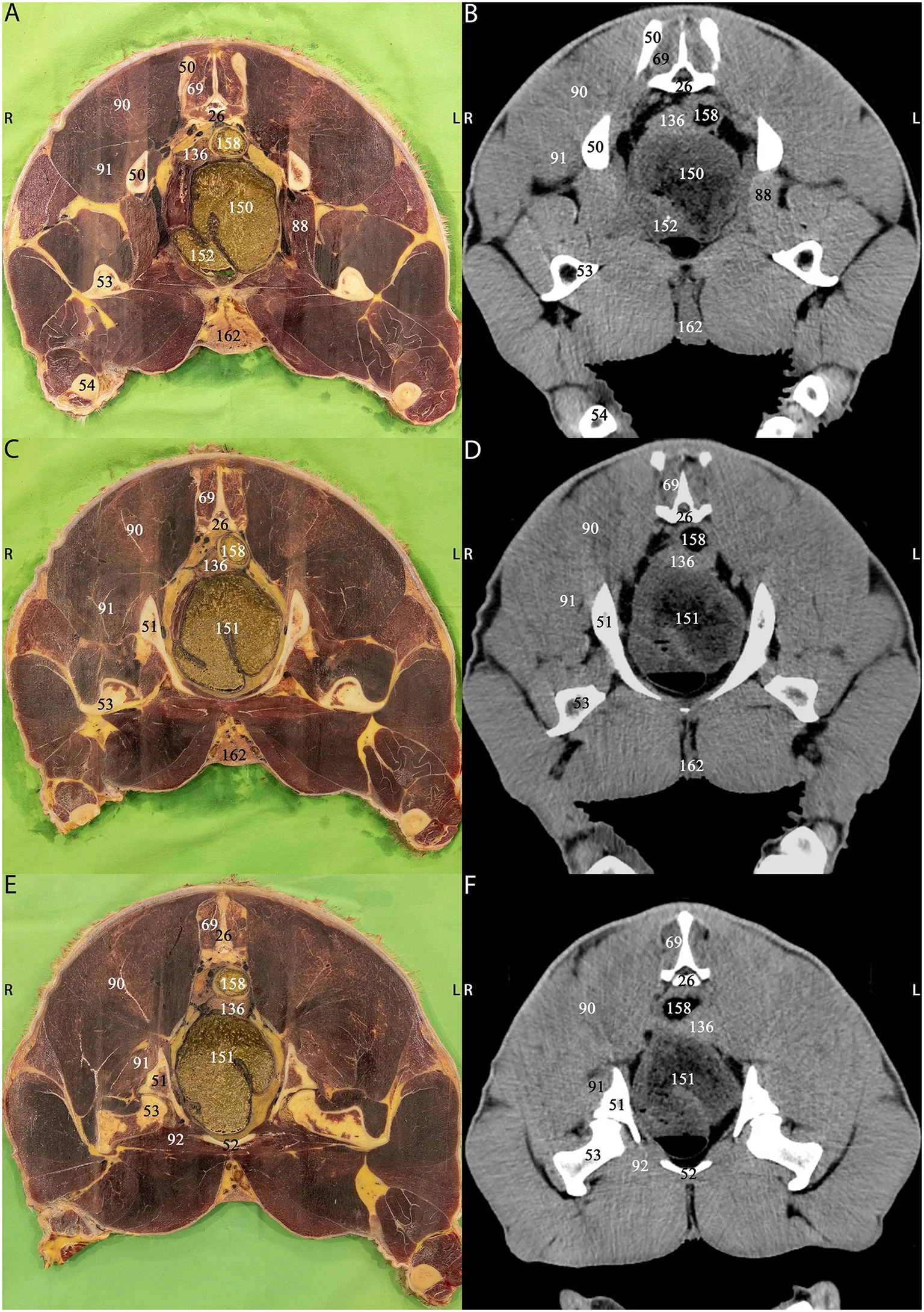
Comparative transverse image at the level of the mid-sacrum. 26. *os sacrum;* 50. *os ilium;* 51. *os ischii;* 52. *os pubis;* 53. *femur;* 54. *tibia;* 69. *m. multifidus;* 88. *m. iliacus;* 90. *m. gluteus medius;* 91. *m. gluteus profundus;* 92. *m. obturatorius internus et externus;* 136. *uterus;* 150. *colon ventrale sinistrum* (left ventral colon); 151. *flexura pelvina* (pelvic flexure); 152. *colon dorsale sinistrum* (left dorsal colon); 158. *rectum;* 162. *glandula mammaria*

**Fig. 16:**
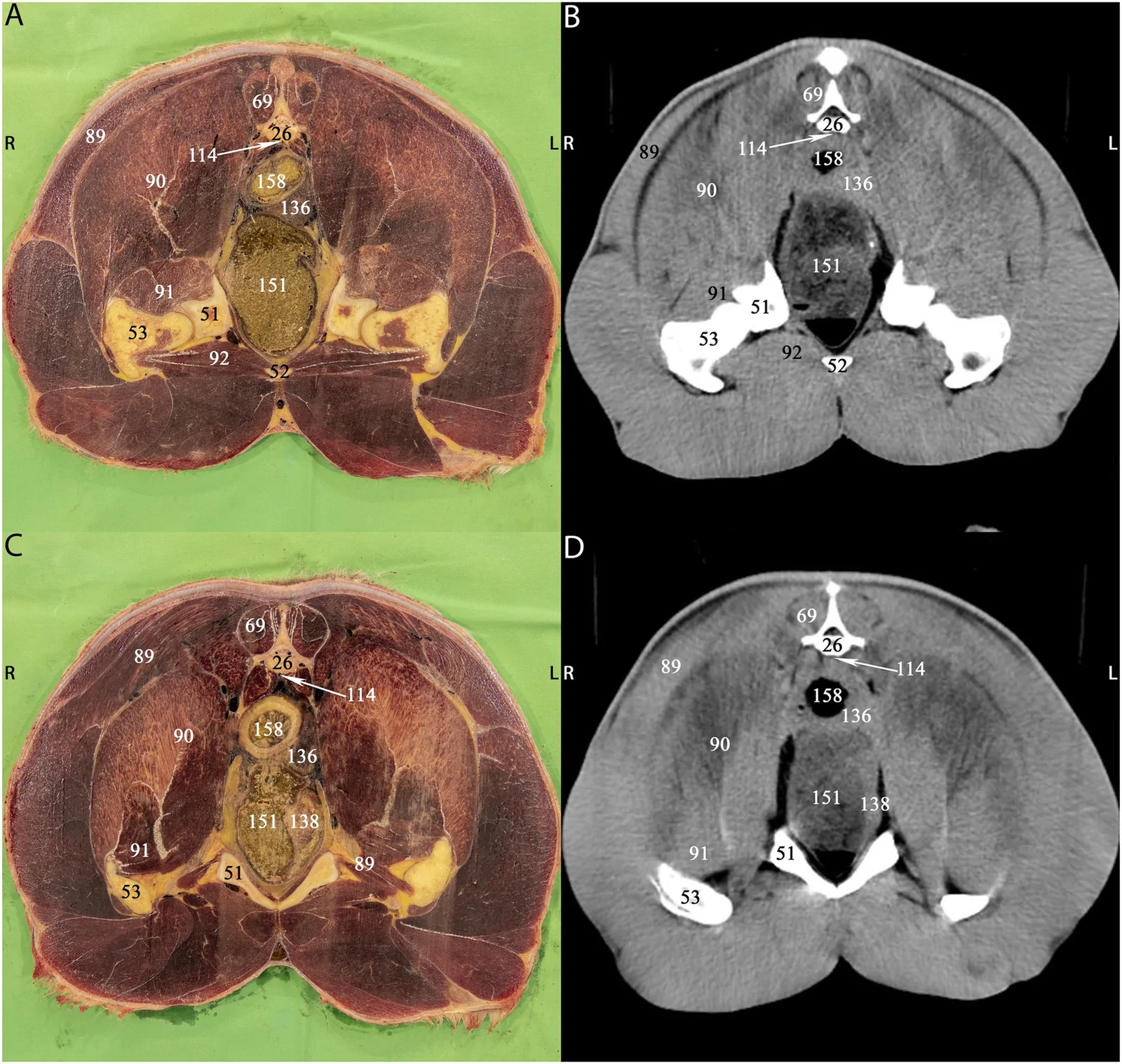
Comparative transverse image at the level of the caudal-sacrum. 26. *os sacrum;* 51. *os ischii;* 52. *os pubis;* 53. *femur;* 69. *m. multifidus;* 89. *m. gluteus superficialis;* 90. *m. gluteus medius;* 91. *m. gluteus profundus;* 92. *m. obturatorius internus et externus;* 114. *a. sacralis mediana;* 136. *uterus;* 138. *vesica urinaria* (bladder); 151. *flexura pelvina* (pelvic flexure); 158. *rectum*

Our comparison of anatomical slices with CT images revealed that perfect alignment between the two could not be achieved. Volume changes caused by freezing and post-euthanasia gas production in the intestinal tract made it difficult to eliminate all discrepancies. However, these differences were minimal and did not significantly affect identification or comparison. Most organs in the anatomical sections were macroscopically visible; however, some smaller structures remained indistinguishable. The images obtained as a result of the work served as a good basis for comparison with the clinical case. Cleaning of the frozen sections with ethyl alcohol resulted in sharp images with high definition (Figs. 1, 2, 3, 4, 5, 6, 7, 8, 9, 10, 11, 12, 13, 14, 15 and 16).

Several major blood vessels were visualised on the CT images. However, most abdominal organ vasculatures, particularly in the mesocolon, were not identifiable. Additionally, the right ovary was not visible, most likely due to its small size and the fact that it was located outside the sectioning plane. When labelling structures, we primarily focused on those identifiable in the CT images, and 163 of them were annotated. Structures with similar radiodensity such as muscles were only distinguishable on CT when interfaced with fatty connective tissue; otherwise, their boundaries were blended. Structures that could not be identified clearly were not labelled. The small intestines, with a few exceptions, proved particularly challenging to differentiate macroscopically. Certain segments, including the descending duodenum and ileum, were defined based on their position [31, 32]. However, a more detailed classification and precise description of transitions between sections would require histological examination or a larger subject.

When identifying the abdominal structures and organs, we concluded that the pelvic flexure was filled with contents and displaced caudally into the bony pelvis; and the apex of the cecum was found at the level of the fourth lumbar vertebra on the left side. Since the pony had had a broken limb for more than 24 hours, we assumed that pelvic flexure impaction had developed as a consequence and was displaced more caudally than the normal anatomical position [31, 32]. The apex ceci, which is normally located at the level of the xiphoid cartilage [31, 32], may have moved during the positioning into dorsal recumbency. We found no data in the literature indicating that the position of the two intestinal segments described here is normal in Shetland ponies.

### Pony no. 2

In this case, CT imaging identified a distended cecum and an enlarged colon filled with large amount of fluid, gas, ingesta with different attenuation values of ingesta, and sand, with no suspicion of any displacement or torsion (Additional files 3, 7). We compared our findings with those seen on the CT images of Pony No. 1. The thickness of the intestinal walls was also reviewed preoperatively, and we found no evidence of any intestinal segment that was clearly thick-walled.

On abdominal CT scans of this horse, the appearance of the large intestinal contents (including gas, fluid, different types of ingesta such as unchewed fibrous intestinal contents, fluid accumulation and sand) varied based on their relative attenuation values and their interaction with X-rays. Gas is hypoattenuating (less dense) compared with soft tissues and has low attenuation values on CT scans (Additional file 3). Marked abdominal distension from fluid- and gas-filled intestinal loops was observed, and resulted in poor differentiation between segments, although they could be partially discriminated based on ventrally settled sand in dorsal recumbency (Additional file 3). Fluid within the intestines is hyperattenuating relative to gas but has lower attenuation values than soft tissues. It may appear as a homogeneous signal, without the granular or fibrous texture observed in the case of unchewed food or ingesta. Because gas accumulates in the non-dependent regions of gastrointestinal tract, gas-fluid levels are visible in the images (Additional file 3, 4, 8).

Ingesta such as partially chewed food is moderately hyperattenuating on CT, and its attenuation values are similar to that of intestinal fluid content. Because of the considerable fluid accumulation in the cecum and the large colon, it was difficult to distinguish the chewed or partially chewed ingesta from the fluid content. Conversely, we could identify moderately hyperattenuating, rounded shaped areas with different attenuation values, reflecting the unprocessed, fibrous nature of the material (Additional file 3).

The HU values were also measured and presented in Additional file 5.

In this particular case, fluid, gas and sand accumulation could be seen, especially in the cecum and large colon (Additional file 4, 8). Sand content appears hyperattenuating on CT; however, it may not be visible unless it is present in large quantities. It tends to settle in the ventral colon and can be observed as a granular pattern on CT images.

To summarize the CT findings, the cecum and large colon were diffusely distended with homogeneous material consistent with fluid and gas accumulation without any displacement or torsion. Different types of ingesta such as unchewed fibrous intestinal contents, ingesta together with fluid accumulation, and large amount of hyperattenuating content consistent with sand were observed in the left dorsal colon (Additional file 9). No evidence of any clearly thick-walled intestinal segment was found, but distinguishing the intestinal wall from the fluid-rich content on the native images is quite challenging, which constitutes the limitation of the technique. Adjacent organs were mildly displaced, but no evidence of free gas or significant peritoneal fluid was present. These advanced imaging findings are compatible with fluid-rich colonic and caecal impaction.

The preoperative plan was based on the CT findings as well as confirmation by laparotomy. In accordance with the CT findings, thick-walled intestinal segments were not found during surgery. The surgical intervention included the evacuation of the impacted cecum and large colon through enterotomies of the cecum and the pelvic flexure, as originally planned.

No abnormalities were observed in the thoracic CT images. There was no evidence of gravity-dependent atelectasis of the lungs or of motion artefacts. The duration time of the CT was 10 minutes.

## Discussion

CT has become increasingly accessible in equine clinics globally [33], with 90-cm gantry systems suitable for small-to-medium-sized horses [3]. Thoracic and abdominal imaging of large horses is not yet feasible; however, it is expected that this limitation will be overcome in the future [34]. Nonetheless, CT examinations may be useful in small- and medium-sized horses, ponies, and foals [17, 35], in whom rectal examinations are either not feasible or pose an increased risk due to their size.

Having an anatomical reference for the interpretation of CT images is essential because it provides a clear understanding of how normal structures appear, which is key to detecting abnormalities. A reference for normal anatomical features, which is already available for other body regions in horses as well as in other species, allows for the identification of pathological changes [6, 8, 9]. Such a reference for abdominal and thoracic cavities of horses and ponies is not available in the literature. Our original aim to complete a CT examination of the abdominal cavity under physiological conditions was mostly attained. However, the position of the pelvic flexure of the large colon, the apex of the caecum, and the content of the pelvic flexure found in Pony No. 1. did not correspond to what is described in the literature [31, 32] which constitutes a limitation of our study. Nonetheless, we believe that our findings significantly contribute to the characterization of the anatomy and pathological changes in the thoracic and abdominal cavities of ponies.

Scanning an anesthetized horse using CT differs from scanning a cadaver due to physiological and motion-related factors. In living animals, subject movements as well as respiratory and cardiac motion can create artefacts [36]. On the other hand, blood flow in a live horse allows for tissue perfusion and contrast-enhanced imaging [37]. This is not possible in cadavers without resorting artificial perfusion. Safety and handling considerations are significant for anesthetized horses, whereas cadavers can be scanned repeatedly without these concerns. Cadaver images are often sharper and artefact-free, making them ideal for anatomical studies or protocol testing. Live horse scans provide clinically realistic images that reflect true anatomy and pathology, despite potential motion- and physiology-related variability.

The position of the patient—standing versus dorsal recumbency—affects the placement and visibility of abdominal and thoracic organs. In the standing position, gravity causes abdominal organs such as the intestines to settle at a lower position [38]. Laparoscopy in standing horses provides excellent observation in both sides of the abdomen, but it is limited to the most ventral portions [39]. In the standing position, the large colon and cecum normally occupy much of the abdominal cavity and are identified ultrasonographically by their location and appearance [40]. The usefulness of laparoscopy for diagnosis and therapy in horses could be enhanced in dorsal recumbency [41]. The anatomy of the abdomen observed in dorsally recumbent horses during laparoscopy or median laparotomy can be expected to be similar to that observed on CT scans combined with anatomical sections in the present study. The location of the abdominal organs in a horse placed in a dorsal recumbency also depends on other positioning details (horizontal position, or inclination of at least 35° into a head-down or head-up position) [41]. Being aware of how these factors influence the outcome, we believe that our study can be helpful not only for the performance of colic surgeries, but also in the planning of laparoscopic surgeries.

Hounsfield Units on CT help to differentiate bowel lumen contents by providing a measurement of tissue density relative to water. Specific materials, including faeces, blood, food, and medications, have characteristic HU ranges that reflect their composition [28]. In the present study, four different types of content were thus identified and subsequently confirmed by intraoperative findings. Interpretation of the images depends on the shape, location, layering, enhancement patterns, and HU values. HU measurements in combination with the clinical context and visual CT features for accurate identification can be a useful in diagnostics, but the present findings require additional confirmation.

Subjective assessment of sand accumulation on abdominal radiographs is used as a diagnostic aid. Nevertheless low grade sand accumulation in the intestine was found to be a frequent finding in horses [42]. According to these authors, when working up a case with clinical signs from the gastrointestinal tract, low grade sand accumulation should not be considered the cause until other possibilities have been ruled out [42]. A disadvantage of radiographic evaluation of intestinal sand content is that it provides a two-dimensional measurement of a three-dimensional structure [43]. Several radiographic scoring systems have been proposed; however, they are largely subjective and have not been found to be suitable for prospective studies [44, 45]. More recent studies have used sand area in cm^2^, which provides quantitative assessment of sand burden [46–48]. A weight-based cut-off of >21 cm^2^ per 100 kg for the diagnosis of sand enteropathy was determined with a sensitivity of 98.25% and specificity of 92.54% [49]. We propose, that CT scans may also provide more accurate sand detection through cross-sectional imaging that avoids overlap and improves visualisation, but further studies are needed to investigate this hypothesis.

Contrast CT has been reported in arteriograms, venograms, and post-contrast scans of soft tissues in horse extremities, as well as in myelography and arthrography studies of horses [50], but its use in the evaluation of abdominal organs has not been described. In native (non-contrast) CT images obtained in dogs and cats, the intestinal wall appears as a thin structure with soft tissue density, typically without visible internal layering. Wall thickness can be evaluated, but subtle changes such as oedema or early inflammation are difficult to detect [51]. Conversely, contrast-enhanced CT provides improved visualisation of the intestinal wall, particularly highlighting mucosal enhancement. In some cases, partial wall layering can be observed, especially when the bowel is filled with fluid, aiding in the identification of pathological conditions in these species [51]. Contrast enhancement also helps to identify abnormalities such as ischaemia, inflammation, or neoplasia based on wall thickness and enhancement patterns in other species [52, 53].

Ultrasound is the primary tool used to measure intestinal wall thickness in horses, because it is accessible, provides real-time data, and allows for the assessment of both wall thickness and motility. However, the presence of gas, the depth and body size can impose limitations [40]. The intestinal wall is normally very thin in ponies [54].

CT offers precise cross-sectional and three-dimensional measurements. The theoretical resolution of CT images depends on pixel size (FOV/matrix) and slice thickness, which together determine voxel size. Reducing slice thickness and FOV can improve spatial resolution, but it also increases image noise, making the imaging of thin walls particularly challenging [27].

In horses, larger pixels (~1 mm) and a slice thickness of 2 mm result in voxels that are still comparable to the thickness of the wall, leading to partial volume effects. As a result, the intestinal wall appears somewhat blurred, and individual layers remain difficult to distinguish, though resolution is slightly better than with thicker slices. This explains why CT has inherent limitations for evaluating thin bowel walls in large animals, even when contrast enhancement would be used. Further research is needed to confirm the results reported here.

Significant physiological changes can affect image interpretation in horses undergoing thoracic CT in dorsal recumbency under general anaesthesia. The dorsal lung regions frequently exhibit gravity-dependent atelectasis due to the weight of the animal and the reduced ventilation under anaesthesia [55]. The heart and mediastinal structures may shift dorsally and appear slightly compressed or displaced [55, 56]. In cadavers, the absence of ventilation and circulation may lead to more uniform or patchy lung collapse with generally higher lung attenuation. Post-mortem hypostasis, fluid redistribution, and loss of vascular definition are common and can mimic severe pulmonary disease [57, 58]. Overall, CT findings on live animal reflect reversible physiologic effects, whereas the changes observed in cadavers are mostly due to passive post-mortem artefacts. In the second case, the CT examination was performed immediately before the surgery (for colic), and this might be the reason why we could not identify any effect of the anaesthesia on the CT images, as the interval between the induction and the CT scanning of the thorax was very short. Moreover, the position of the pony changed several times, even during this short period.

The Confidential Enquiry into Perioperative Equine Fatalities: phase 4 (CEPEF4) study showed that perioperative mortality in healthy horses undergoing elective general anaesthesia is low (generally less than 2%) [59]. While longer anaesthesia duration is associated with increased risk, this increase is gradual, and short extensions (10 minutes) contribute minimally to overall risk [60]. Modern anaesthesia protocols, continuous monitoring, and experienced teams further mitigate complications during elective procedures. Emergency cases or horses with comorbidities carry higher risk, highlighting the fact that controlled settings are important to minimise adverse outcomes. Therefore, a brief extension under anaesthesia in a monitored, elective surgery context is generally considered to be low risk [61]. The use of data obtained from the first case as a reference was valuable to understand the CT images obtained from the second (clinical) case and formulate an appropriate surgical plan. Without this aid and previous experience, the evaluation of the images could have been confusing and could have been potentially misinterpreted.

The use of abdominal and thoracic CT in horse is advancing rapidly due to the development of large-bore scanning equipment, which make cross-sectional imaging more accessible. Overall, abdominal CT may become a key tool in equine diagnosis and surgery, improving clinical outcomes and expanding possibilities in both research and practice. Future studies should include stallions and geldings to account for anatomical variations in the pelvic structure. Additional studies supplemented by clinical case reports are necessary to obtain a more accurate understanding of age-, breed-, and sex-based differences. Although intravenous contrast may improve visualisation of vessels and the intestinal segment in equine abdominal CT, but in large adult horses, the limited spatial resolution of the CT images might prevent the visualisation of small vessels. Our results provide a good reason for optimism regarding CT examination of the equine thorax and abdominal cavity; however, in addition to anatomical references, studies including a higher number of individuals are also necessary to confirm the clinical application of these findings.

## Conclusions

In our work on two ponies we have shown two different aspects on the usefulness of CT in equine medicine. In the case of the first pony the comparison of the transverse CT slices with the anatomical structures seen on the immediate post-mortem slices was clearly possible, allowing us to create a reference that was previously unavailable. Moreover, this work made it possible to label the structures in the CT image, and 163 of them were annotated.

The second pony was a clinical case of colic where the CT imaging proved to be valuable in the preoperative assessment of the abdominal situation, identifying the distension of the cecum and large colon with fluid, gas, ingesta and sand. In this case the preoperative plan was based on the CT findings as well as on the corresponding findings at laparotomy. Moreover, we could use the results of the first pony’s anatomical and CT findings as a reference, they were great help in planning the colic surgery in the second pony.

In conclusion, the combination of anatomical and CT imaging provides complementary information, thus enhancing the identification of normal and pathological abdominal structures.

## Electronic supplementary material

Below is the link to the electronic supplementary material.

Supplementary material 1: CT examination of the cadaver pony (12-year-old mare, weight: 130 kg). The body was scanned in two sections with a dorsally recumbent 70 cm field of view (FOV)

Supplementary material 2: Preoperative laterolateral radiograph of the ventral abdomen of the pony which underwent colic surgery

Supplementary material 3: Transverse, sagittal and dorsal (Th9) views. Note the different types of ingesta such as unchewed fibrous intestinal contents, ingesta together with fluid accumulation, and large amount of hyperattenuating content consistent with sand in the left dorsal colon. The right side corresponds to the left portion of the image. 1. Th9, 2. aorta, 3. v. cava caudalis, 4. liver, 5. diaphragmatic flexure, 6. sternal flexure, 7. cecum, a. gas in the diaphragmatic flexure, sternal flexure and cecum, b. fluid content in the sternal flexure and cecum, c. moderately dense, rounded shape composed of areas of different density, reflecting the unprocessed, fibrous nature of the material different types of ingesta such as unchewed fibrous intestinal contents in the diaphragmatic flexure, d. sand in the diaphragmatic flexure, sternal flexure and cecum

Supplementary material 4: Transverse CT scan at the level of Th17. Note the different types of ingesta such ingesta together with fluid accumulation, and the large amount of hyperattenuating content consistent with sand in the left dorsal colon. The right side corresponds to the left portion of the image. 1. Th17, 2. right kidney, 3. head of the cecum, 4. ampulla coli (right dorsal colon), 5. right ventral colon, 6. apex of the cecum, 7., left ventral colon, 8. spleen, 9. left dorsal colon, 10. left kidney, a. hypoattenuating gas accumulation in the head of the cecum, right ventral colon, apex of the cecum and the left ventral colon, b. large amount of fluid content in the head of the cecum, ampulla coli, right ventral colon, apex of the cecum, left ventral colon and left dorsal colon, d. sand in the apex of the cecum, left ventral colon and left dorsal colon

Supplementary material 5: Hounsfield Unit (HU) values of different intestinal contents and parenchymal abdominal organs

Supplementary material 6: Pelvic flexure enterotomy. Evacuation of the large colon through pelvic flexure enterotomy (9-year-old pony mare, weight: 125 kg). Different types of ingesta, including unchewed fibrous intestinal contents, ingesta with fluid accumulation, and sand were observed

Supplementary material 7: Transverse CT scan at the level of L2. Note the fluid accumulation in the large colon, and the extreme amount of fluid content in the caecum. The right side corresponds to the left portion of the image. 1. L2, 2. cecum, 3. left ventral colon, 4. left dorsal colon, 5. left kidney, 6. small colon, 7. spleen, a. gas in the cecum and left ventral colon, b. large amount of fluid content in the cecum, left dorsal colon and left ventral colon

Supplementary material 8: Transverse CT scan at the level of Th15. Note the different types of ingesta such ingesta together with fluid accumulation, and large amount of hyperattenuating content consistent with sand in the left dorsal colon. The right side corresponds to the left portion of the image. 1. Th15, 2. liver, 3. head of the cecum, 4. right dorsal colon, 5. right ventral colon, 6. apex of the cecum, 7. left ventral colon, 8. spleen, 9. left dorsal colon, 10. stomach, a. gas in the head of the cecum, right ventral colon, apex of the cecum and the left ventral colon, b. fluid content in the head of the cecum, right ventral colon, apex of the cecum and the left ventral colon, c. different densities of ingesta in the right dorsal colon and the left dorsal colon, d. sand in the apex of the cecum and the left ventral colon

Supplementary material 9: 3D reconstruction of the sand content in the abdomen. a: left lateral view, b: caudal view (the right side corresponds to the left portion of the image)

## Data Availability

The datasets used and/or analysed during the current study are available from the corresponding author on reasonable request.

## Abbreviations

CT: Computed tomography
HU: Hounsfield Unit
FOV: Field of view
MRI: Magnetic resonance imaging
WW: Window width
WL: Window length
ROI: Region of interest

## Declarations

### Prior publication

Data have not been published previously.
