# Supplementary material for: Computed tomography anatomy of the pony thorax and abdomen and an example of a clinical application
Source: Acta Vet Scand. 2026 Nov 3;68:48. doi: 10.1186/s13028-026-00891-4 (PMC13729400; doi:10.1186/s13028-026-00891-4)
Supplement: Supplementary file 1 [file 13028_2026_891_MOESM5_ESM.docx]

**Supplementary Table 1.** Hounsfield Unit (HU) values and macroscopic appearance of different intestinal contents and parenchymal abdominal organs. On Supplementary Figure 5. the ROIs were made freehand, and on Supplementary Figure 7. it was made with circular. The Mean HU values were recorded together with the standard deviations.

|  | HU values (Mean +/- SD) | Macroscopic appearence | ROI size (mm^2^)/ pixels |
| --- | --- | --- | --- |
| Sup. Fig 5. flex. sternalis fluid content | 16.89+/-10.59 | fuid content | 2280.34/54x54 |
| Sup. Fig 5. flex sternalis gas content | -992.35+/4.47 | gas | 1220.28/39x39 |
| Sup. Fig 5. liver | 66.35+/-7.99 | normal | 588.00/588 |
| Sup. Fig 5. flex. diaphragmatica sand | 587.42+/-143.23 | significant amount of gray sand | 793/793 |
| Sup. Fig 5. flex. diaphragmatica unchewed content | -139.90+/66.63 | unchewed fibrous material | 620/620 |
| Sup. Fig 7. Cecum fluid content | 11.51+/-11.39 | fluid content | 1612.72/ 45x45 |
| Sup. Fig 7. cecum (apex) sand content | 768.28+/-126.73 | significant amount of gray sand | 141.56/13x13 |
| Sup. Fig 7. ampulla coli fluid | 9.70+/14.19 | fluid content | 2099.48/52x52 |
| Sup. Fig 7. left dorsal colon fluid | 14.21+/-16.31 | fluid content | 576.05/27x27 |
| Sup. Fig 7. left ventral colon mixed content | 63.68+/-37.18 | fluid ingesta and sand | 2099.48/52x52 |
| Sup. Fig 7. left ventral colon fluid content | 24.47+/-11.67 | fluid content | 2099.48/52x52 |
| Sup. Fig 7. right kidney | 38.09+/-14.67 | normal palpation | 830.04/33x33 |
| Sup. Fig 7. right ventral colon fluid content | 19.67+/-13.85 | fluid content | 2099.48/52x52 |
